# Substructure-Specific Antibodies Against Fentanyl Derivatives

**DOI:** 10.1021/acsnano.4c14369

**Published:** 2025-01-10

**Authors:** Asheley Chapman, Minghao Xu, Michelle Schroeder, Jason M. Goldstein, Asiya Chida, Joo R. Lee, Xiaoling Tang, Rebekah E. Wharton, M. G. Finn

**Affiliations:** † School of Chemistry and Biochemistry, Georgia Institute of Technology, 901 Atlantic Dr., Atlanta, Georgia 30332, United States; ‡ Immunodiagnostic Development Team, Preparedness, Response, & Outbreak Services Branch, Division of Core Laboratory Services & Response, Office of Laboratory Systems and Response, Centers for Disease Control and Prevention, 1600 Clifton Rd NE., Atlanta, Georgia 30333, United States; § Division of Laboratory Sciences, National Center for Environmental Health, Centers for Disease Control and Prevention, 4770 Buford Hwy, Atlanta, Georgia 30341, United States; ∥ School of Biological Sciences, Georgia Institute of Technology, 901 Atlantic Dr. Atlanta, Georgia 30332, United States

**Keywords:** fentanyl derivatives, antibodies, immune response, diagnostics, virus-like particles, immunization

## Abstract

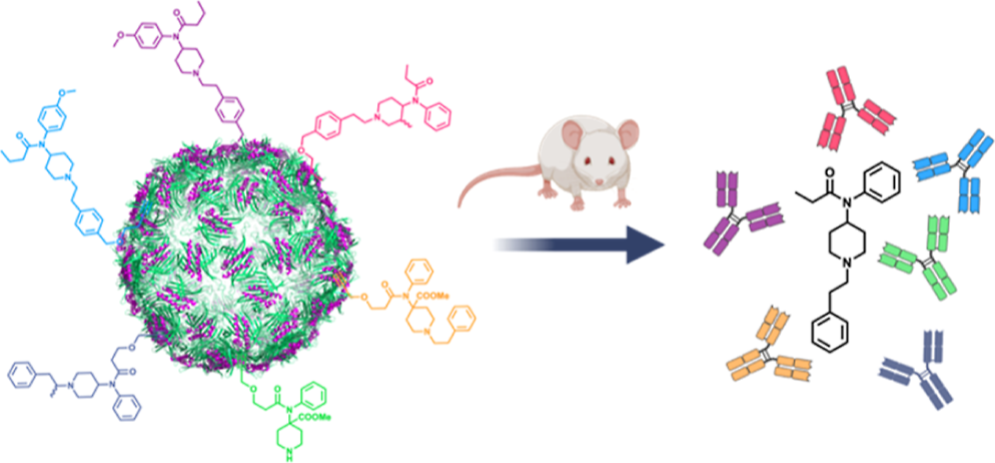

Structural variants of the synthetic opioid fentanyl are a major threat to public health. Following an investigation showing that many derivatives are poorly detected by commercial lateral flow and related assays, we created hapten conjugate vaccines using an immunogenic virus-like particle carrier and eight synthetic fentanyl derivatives designed to mimic the structural features of several of the more dangerous analogues. Immunization of mice elicited strong antihapten humoral responses, allowing the screening of hundreds of hapten-specific hybridomas for binding strength and specificity. A panel of 13 monoclonal IgG antibodies were selected, each showing a different pattern of recognition of fentanyl structural variations, and all proving to be highly efficient at capturing parent fentanyl compounds in competition ELISA experiments. These results provide antibody reagents for assay development as well as a demonstration of the power of the immune system to create binding agents capable of both broad and specific recognition of small-molecule targets.

The opioid overdose epidemic remains a critical public health epidemic in the United States, with nonfatal^1^ and fatal overdoses increasing steadily since 2011 and claiming a staggering 50,000–80,000 lives each year since 2016.^2^ In 2022, 75% of overdose deaths captured in the CDC’s State Unintentional Drug Overdose Reporting System involved illegally made fentanyl.^3^ Among the most active μ-opioid receptor agonists, fentanyl is 10 times more lethal than heroin and up to 1000 times more potent than morphine (Table 1), with the analogue carfentanil ten times more potent still.^4−9^ First discovered in 1960 and approved as an intravenous anesthetic in 1972, reports of misuse by surgeons and anesthesiologists with access to the drug appeared in the 1980s, followed by overdoses in patients and the wider population after transdermal fentanyl was approved for pain management in the 1990s.^10^ In the U.S., fentanyl emerged on the black market as a filler in illicit heroin, and is now also found in other illegal drugs like cocaine, counterfeit pharmaceuticals, and as a single source narcotic.^11−14^

**Table 1:** Relative Potency and Lethality of Opioids, Including Fentanyl Derivatives^a^

opioid	ED_50_ (mg/kg)	LD_50_ (mg/kg)	relative potency	relative lethality
morphine	3.21^6^	223^6,15^	1	1
heroin	0.129^16^	22.5^7,15^	25	10
fentanyl	0.0037–0.011^8^	3.05^5^	823	73
carfentanil	0.0004^9^	3.39^5^	8025	67
3-methyl fentanyl^b^	0.00058^17^		5534	
α-methylacetyl fentanyl^c^	0.106^18^		30	

a All measurements taken in Sprague-Dawley rats after intravenous administration and determined by similar methods.

b Mixture of *cis-* and *trans*-isomers.

c Peroral administration.

Due to the influx of illicitly synthesized fentanyl analogs,^19,20^ detection and subsequent control of these substances has also become increasingly difficult. Law enforcement and emergency responders that encounter overdose victims or illegal drugs in the field would benefit from reliable, rapid, inexpensive, and easily interpreted assays to better treat patients, protect themselves from exposure, and trace the origins of the hazardous compounds. Lack of identification impacts fentanyl analogue regulation, prosecution of producers and distributors, and effective treatment of those affected by their use. Diagnostic tools and treatments are therefore needed to identify these compounds in a variety of settings and thereby prevent deaths.

While many techniques are available for fentanyl detection including mass spectrometry and other analytical methods, immunoassays using antifentanyl monoclonal antibodies (mAbs), such as enzyme-linked immunosorbent assays (ELISA) and lateral flow assays (LFA), are among the most widely employed for reasons of convenience, speed, and cost.^17,21−23^ Deployed as point-of-care harm reduction tools, fentanyl immunoassays available to users can allow immediate detection of such compounds in various drugs of abuse and allow users to mitigate risk of unwitting overdose.^24^ A study by Wharton et al. in 2021 summarized the diagnostic coverage of more than 30 commonly encountered fentanyl derivatives by 19 commercially available immunoassays.^18^ It was found that, while most tests are adept at identifying one or a few compounds, no test is yet capable of sensitively detecting a significant fraction of the total. Importantly, certain potent analogues, such as 3-methylfentanyl, are poorly detected by all current commercial immunoassays and may therefore be diagnostically underreported.^18,25−29^ Similar results for a smaller set of derivatives were reported with a commercial assay kit using a single antibody, adding observations that fentanyl could be distinguished from heroin and other neurologically active drugs.^25^

The quality of immunoassays depends on the specificity and affinity of the receptor molecule (usually a monoclonal antibody) for the target; mAbs able to distinguish among the many fentanyl variants have not been reported. Indeed, developing such binders can be challenging. Small molecules like fentanyl do not bind major histocompatibility complex (MHC) molecules and so are not presented to T cells by MHC-bearing antigen presenting cells, an essential step in the formation of antigen-specific immunoglobulin G (IgG) antibodies. As such, mAbs against small molecules are typically produced with vaccines formed by attaching the target drug molecule (a hapten) to an immunogenic carrier protein.

This approach has a long history^30^ and has been most enthusiastically advanced for fentanyls and other drugs of abuse by the groups of Janda,^16,31−36^ Pravetoni,^37−39^ and others. Conjugate vaccines employing carrier proteins KLH, ovalbumin, BSA, CRM_197_, and tetanus toxoid covalently displaying synthetic fentanyls all have been shown to produce antinociceptive effects and prophylactic protection from fentanyl overdose in animals,^16,32,37,40,41^ including nonhuman primates.^40,42^ While effective, the use of globular proteins as carriers for hapten vaccines has its limitations, including poor characterization, suboptimal antigen presentation, and inferior lymph node trafficking.^43^

To isolate high-affinity antibodies sensitive to variants of the fentanyl structure, we used virus-like particles (VLPs) instead. VLPs are recombinant self-assembling protein nanoparticles with size, multivalency, and adjuvating properties that make them ideal carriers for small-molecule haptens.^43^ Pathogen-derived VLPs are in use in the clinic against HPV^44,45^ and HepB,^46^ and many other VLP-based immunogens have been explored over more than two decades against a litany of targets.^47−49^ The VLPs based on the structurally related bacteriophages Qβ and PP7 have been shown to produce IgG in the context of other psychoactive haptens such as nicotine,^50,51^ even advancing to Phase II clinical trials.^52^ Antinicotine antibody titers in these experiments were consistently strong and persistent, although these candidates did not advance to clinical use due to lack of evidence of smoking cessation by vaccinated individuals.^52^

In this study, we created a panel of mAbs by VLP-hapten immunization in mice capable of distinguishing between the parent fentanyl and seven analogues representing different structural classes that are not well-detected by current immunoassays.^18^ Each analogue was equipped with a terminal alkyne through a short flexible linker, enabling conjugation via click chemistry to PP7 VLPs bearing a large number of azide groups. Eight immunogens were created and used to immunize BALB/c mice. High-throughput hybridoma production gave rise to hundreds of candidate antifentanyl mAbs, which were characterized for antigen specificity and fentanyl cross-reactivity. Ultimately, 13 mAbs were identified and brought forward as potential diagnostic reagents that, in various combinations, are capable of discriminating among and between most of the common fentanyl analogues via immunoassay. These reagents can help address a critical blind spot in the tools available for field and laboratory detection of harmful compounds in the opioid epidemic.

## Results/Discussion

### Hapten Design and Vaccine Assembly

**Figure 1:**
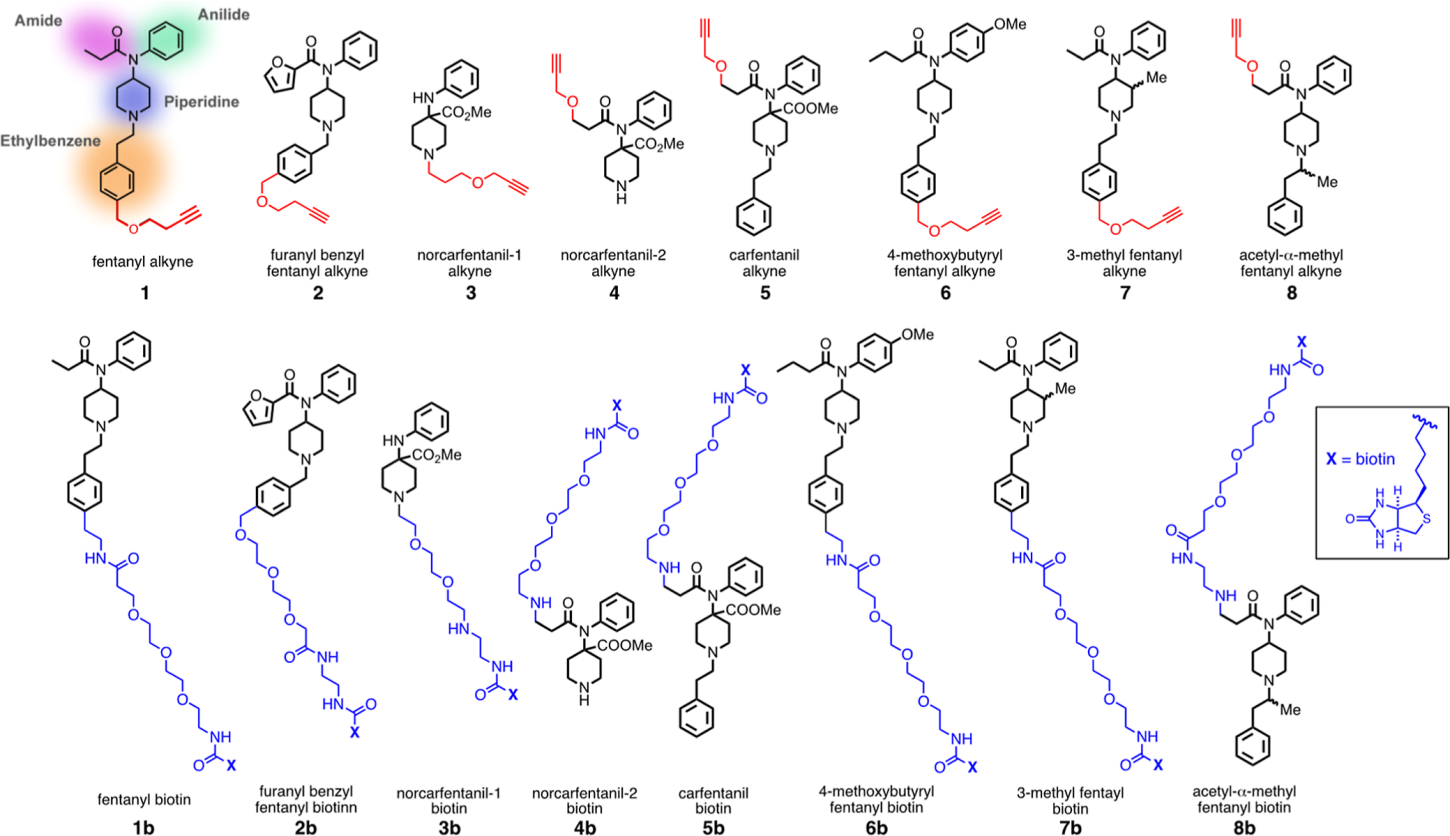
(top) Alkynes of fentanyl-based structures used for conjugation to VLP-azides to construct immunogens for these studies. Compound **1** shows the components of the fentanyl structure. (bottom) Biotinylated reagents for characterization of antibody binding using different linkers than in the immunogens.

In addition to the parent fentanyl structure, we identified six derivatives–carfentanil, norcarfentanil (the major and nontoxic metabolite of carfentanil), 4-methoxybutyrylfentanyl, 3-methylfentanyl, acetyl-α-methyl fentanyl, and 2-furanylbenzyl fentanyl–as being a structurally diverse representation of the fentanyl analogs found in actual drug seizures by the United States Drug Enforcement Agency (DEA) in recent years and identified in the National Forensic Laboratory Information System (NFLIS).^21,53^ One structure, 2-furanylbenzyl fentanyl, was designed as a combination of furanyl- and benzyl-fentanyl derivatives. We chose the amide and ethylbenzene portions of the structure (Figure 1) as connecting points for the alkyne-terminated linkers required to tether the molecules to immunogenic carrier protein nanoparticles by copper-catalyzed azide–alkyne cycloaddition (CuAAC). Our assumption, consistent with reports of Janda and others,^54,55^ is that immune recognition of the hapten alone is most likely to occur to parts of the structure farthest from the linker. Therefore, the smallest target, norcarfentanil, was converted to two different haptens, differing in the position of the linker: on the “tail” (the para position of the ethylbenzene ring, **3**) or the “head” (amide substituent, **4**) of the structure. A corresponding biotinylated derivative of each hapten was made for ELISA analysis of immune response; each structure incorporated a different linker than the immunogen to eliminate the possibility of an antilinker response being mistaken for hapten recognition.

**Figure 2:**
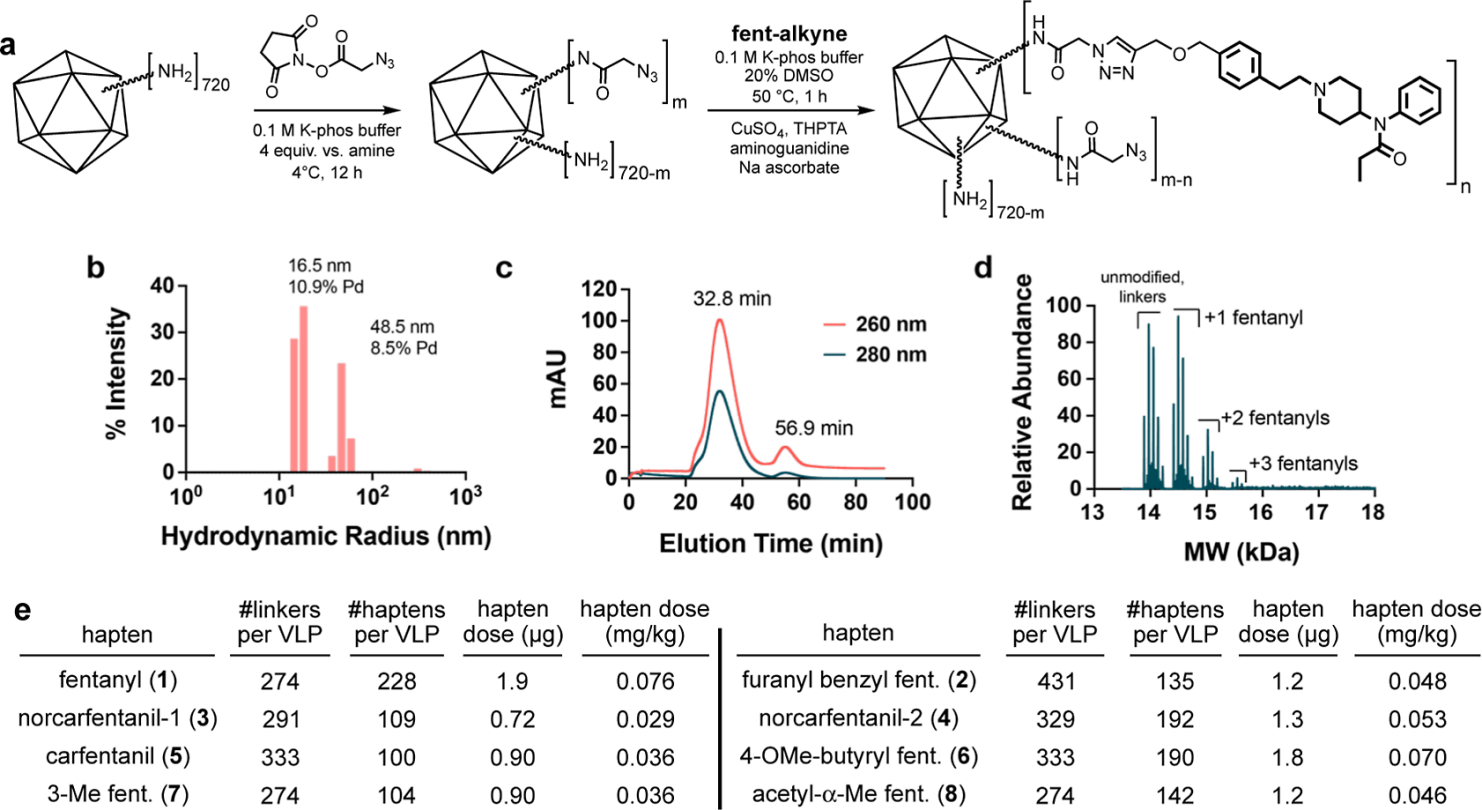
VLP-fentanyl conjugate vaccine design and characterization. (a) Bioconjugation scheme for VLP-conjugates. (b–d) Representative characterization data for VLP-fentanyl conjugates (furanyl benzyl fentanyl [2] shown), (b) dynamic light scattering; pd = dispersity of the indicated cluster of peaks. (c) Size-exclusion chromatography (Superose 6). (d) Mass spectrometry of denatured particle showing distribution of coat protein substitution. (e) Summary of functionalization parameters for the indicated vaccines (±10%). Hapten dose = the amount of fentanyl analog delivered in each 50 μg dose of VLP conjugate; mg/kg calculated for mouse weight of 25 g.

Icosahedral^56^ VLPs derived from the PP7 bacteriophage were recombinantly produced in *E. coli* and characterized by size-exclusion chromatography and dynamic light scattering as previously described.^57,58^ These particles were acylated using azidoacetic *N*-hydroxysuccinimide ester and then addressed with alkynyl fentanyl derivatives by CuAAC conjugation to provide VLP-fentanyl conjugate hapten vaccines (Figure 2a). Mass spectrometry was used to quantify the average number of linkers and antigens attached to coat protein monomers in each step (Figures 2d and S1), assuming equal sensitivity toward modified and unmodified proteins; the average numbers of linkers and haptens per particle were obtained by multiplying this value by 180 coat proteins per capsid. Average loadings of fentanyl-based antigens were found to be between 100 and 230 per particle. These hapten-decorated particles exhibited a modest amount of aggregation in dynamic light scattering (Figures 2b and S2) and in size-exclusion chromatography (small shoulder at shorter retention time, Figures 2c and S3), presumably due to the hydrophobic nature of the attached fentanyl derivatives. However, no precipitation was observed and recovered yields remained good (74–95%). Higher densities of attachment did induce irreversible precipitation, and so were avoided.

### Immune Response

**Figure 3:**
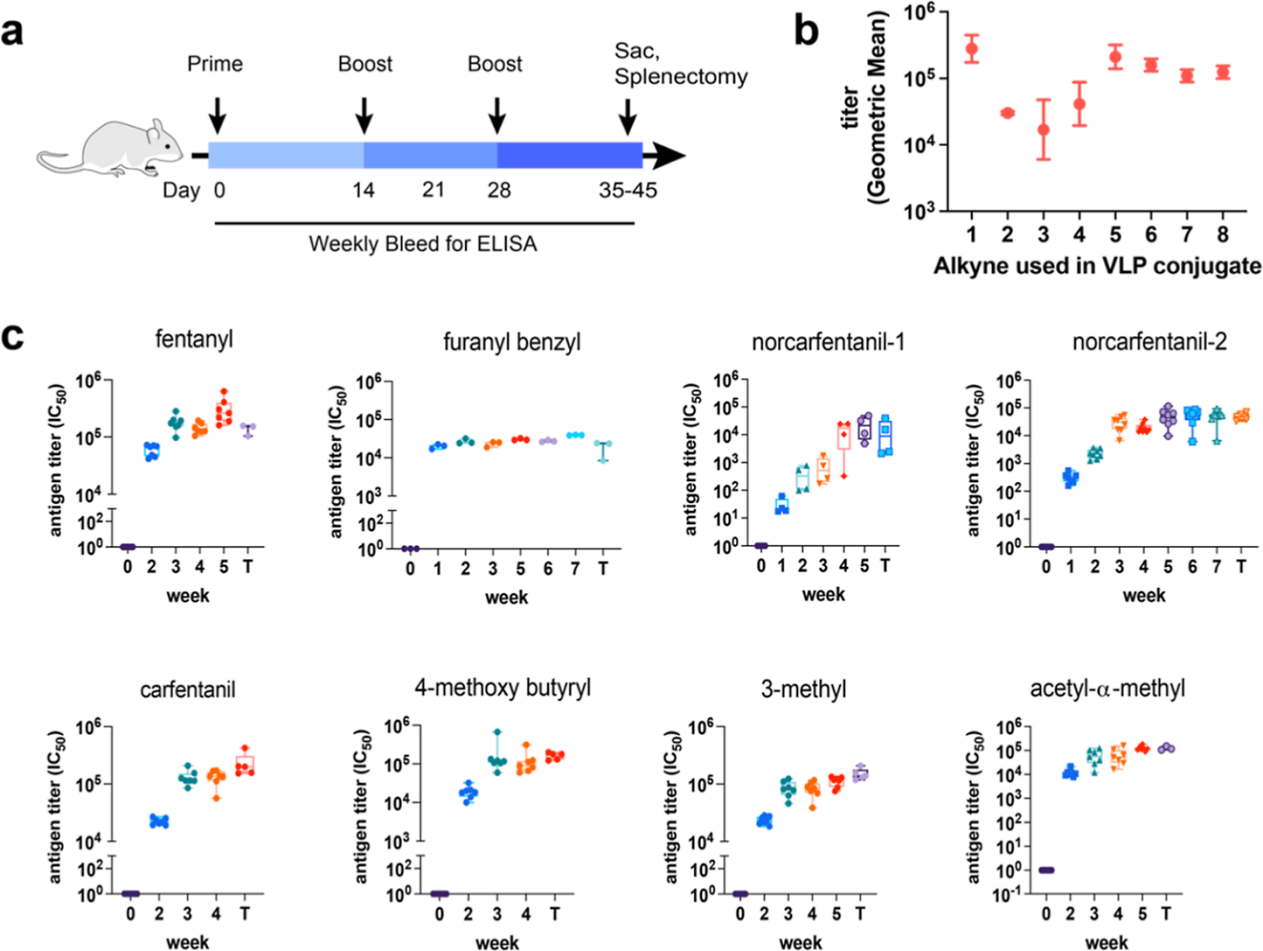
Immunogenicity of PP7-fentanyl conjugate vaccines. (a) Representative vaccine schedule of BALB/c mice immunized with 50 μg of PP7-fentanyl conjugates (500 ng of NKT adjuvant included in prime and final boost) (carfentanil and norcarfentanil-2 terminated day 50; norcarfentanil-1 and 4-MeO-Bu terminated on 35). (b) Peak antihapten titer (week 5 postprime, geometric mean ± 95% CI) for each PP7-fentanyl vaccine. (c) Antifentanyl IgG titer of mice immunized with PP7-fentanyl vaccines as described in Figure 2. The last time point (*T*) represents the terminal bleed. All ELISA assays shown here were performed against the corresponding biotinylated antigen. For example, immune response against the VLP bearing fentanyl alkyne **1** was assayed by ELISA against biotinylated fentanyl **1b.** Titers calculated as IC_50_ by nonlinear regression of 6-fold 5x serum dilutions against specific immunogen by ELISA. Titers reported as box and whiskers ± min/max, line at mean. *n* = 3–7 mice per group.

BALB/c mice were immunized, boosted twice (days 14 and 28), and given a final stimulating boost (1/5th dose) 3 days prior to harvest (Figure 3a). All doses were 50 μg of particle protein, each delivering 0.035–0.1 mg/kg fentanyl derivative (Figure 2e), almost 3 orders of magnitude less than the reported LD_50_ of the parent fentanyl molecule. While VLPs are considered self-adjuvanting because they encapsulate expression host bacterial mRNA during assembly which act as Toll like receptor 7/8 ligands,^59^ the prime and final boost doses both included 500 ng of PBS-57, an α-galactosyl ceramide derivative specific for NKT cell activation (and thus designated “NKT”),^60^ as described in Supporting Information. We observed no toxicity, as indicated by steady weight gain in immunized mice (Figure S5). Note that neither the potency nor toxicity of the alkyne-functionalized fentanyl analogs used here are known but are likely to vary substantially based upon linker position on the fentanyl scaffold. Fentanyl potency is related to μ-opioid receptor affinity as well as to the molecular conformation; both the *N*-phenylethyl and *N*-phenylpropanimide groups have been shown to participate in binding to the receptor pocket.^61,62^

Antifentanyl titers were measured in immunized sera by ELISA using biotinylated fentanyl molecules modified at the same position as the hapten (Figure 1, bottom). Each VLP-fentanyl vaccine produced high antifentanyl titers after one immunization that remained high until end point (Figure 3b,c) although a subsequent two boosts were given to continue to bolster IgG production. While target serum response was robust among all vaccines, immunizations with furanyl benzyl fentanyl (**2**) and alkynes derived from the smaller norcarfentanil metabolites (**3** and **4**) resulted in peak titers (week 5 postprimary immunization) 10-fold lower than other analogues and fewer antigen-specific mAbs (see below).

It is not uncommon in the case of carbohydrate haptens to observe antigen-specific IgM production.^63^ IgM production hints at T-independent antibody generation achieved through B cell receptor cross-linking mediated by multivalent antigen display, undesirable as CD4+ T cell help is a requirement for high-affinity IgG production. Similarly, for cocaine, the production of IgM can significantly diminish antitarget IgG and vaccine efficacy.^64^ We found most of the vaccines tested here to produce very low IgM response peaking at week 2 (Figure S6) when IgM is most expected early in the vaccine schedule.

**Figure 4:**
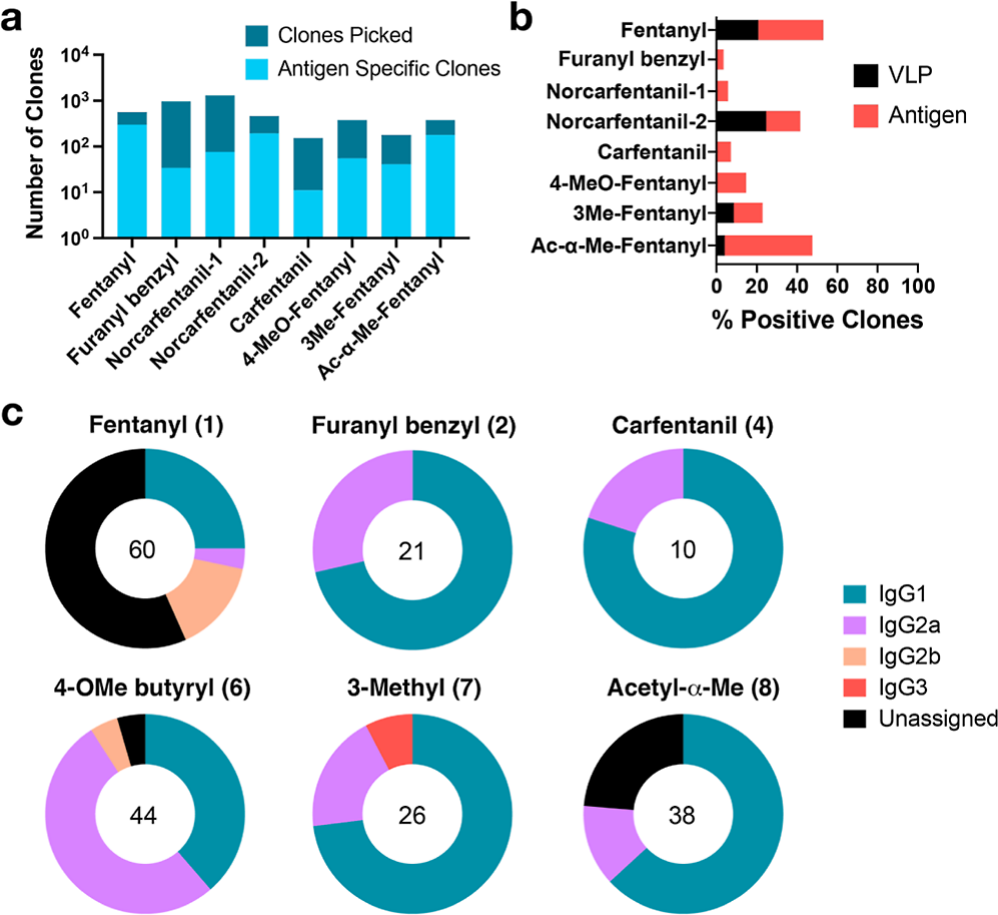
Characterization of antifentanyl hybridomas. (a) Hybridoma clones selected from each PP7-fentanyl immunization (dark blue) and number of antigen-specific clones from this population (light blue). Clones chosen by ClonePix based on morphology and degree of IgG-secretion measured by anti-IgG FITC. Antigen specificity determined by binding to the corresponding biotinylated immunogen via ELISA. (b) red = percent of hapten-specific clones from panel a (light blue/dark blue); black = percent of hapten-binding IgG clones that also bound VLPs. (c) Antifentanyl IgG subclass distribution of hybridoma-produced mAbs from six PP7-fentanyl immunizations. Clones derived from immunization with norcarfentanyl derivatives **3** and **4** are not included here and were not carried forward.

Mice having the highest serum titers of antifentanyl IgG in each vaccine group were selected for mAb production. Hybridomas were formed by electrofusion of spleen-derived B cells with myeloma cells in 3D semisolid culture. Clones secreting IgG were selected and tested for antigen specificity by ELISA. More than 4500 hybridomas were screened for fentanyl specificity, and using this high-throughput approach, we identified 851 antigen specific clones (Figure 4a). The highest proportion of antigen-specific IgG secreters came from immunizations of fentanyl (**1**), norcarfentanil-2 (**4**), and acetyl-α-methyl fentanyl (**8**) (>40% of clones) while much lower fractions of antigen-specific clones came from the other VLP-displayed haptens. We also found no recognition of soluble naloxone by polyclonal sera derived from VLP-fentanyl immunization by competition ELISA (Figure S7).

Of antigen-specific clones, a variable but minor fraction (0–25%) were found by ELISA to also bind the PP7 capsid (Figures 4b and S10). For only one immunogen (the small norcarfentanil-2 structure) was cross-reactive response to the immunogenic carrier found for more than half of the hybridomas analyzed. Simultaneous recognition of both hapten and protein could indicate the existence of “combination” epitopes composed of elements of both, or recognition of a structural motif common to both. This does not include the triazole moiety, since all of the biotinylated reagents used in ELISA assays were made without triazoles in the linker. Subsequent experiments described below were performed with clones that did not cross-react with carrier protein, and all clones from immunization with the smallest haptens (both norcarfentanils) were also excluded. In supernatants from immunizations of six fentanyl derivatives (fentanyl, furanyl benzyl, carfentanil-1, 4-methoxy butyryl, 3-methyl, and acetyl-α-methyl fentanyl), IgG1 was the predominant subclass in all formulations, with IgG2a a close second indicating T-cell help and the kind of mixed Th1/Th2 cytokine milieux typical of protein-hapten conjugate vaccines (Figure 4c).^65,66^

### Antigen-Specific Binding

To survey the potential for specific recognition of fentanyl derivatives, 21 antigen-specific monoclonal antibodies (mAbs) from immunization with furanyl benzyl fentanyl (**2**) were purified from cultured hybridomas and tested for cross-reactivity against 28 unmodified fentanyl analogs by competition ELISA against the biotinylated furanyl benzyl reagent **10** at 50-fold lower concentration (Figure S8). All of the 21 mAbs bound the cognate furanyl benzyl fentanyl agent, illustrating the effectiveness of the hapten strategy, and also recognized benzyl fentanyl, and (to a lesser extent) the parent fentanyl and *o*-fluoro fentanyl. None of the antibodies were able to competitively bind the compounds containing the tertiary ester substituent on the piperidine ring characteristic of carfentanil structures, nor were the truncated *N*-benzylpiperidines NPP, 4-ANPP, and desproprionyl-*p*-fluorophenyl fentanyl recognized by this panel. Thus, both the piperidine + amide “head group” and the benzylic “tail” were both recognized by monoclonal antibodies elicited by the VLP-**2** vaccine. Furthermore, the addition of the quaternary methyl ester substituent of the carfentanils or a methoxy substituent on the aniline fragment abrogated binding by this entire family of mAbs, suggesting that a flat hydrophobic pocket may be a common feature to accommodate the target structure.

**Figure 5:**
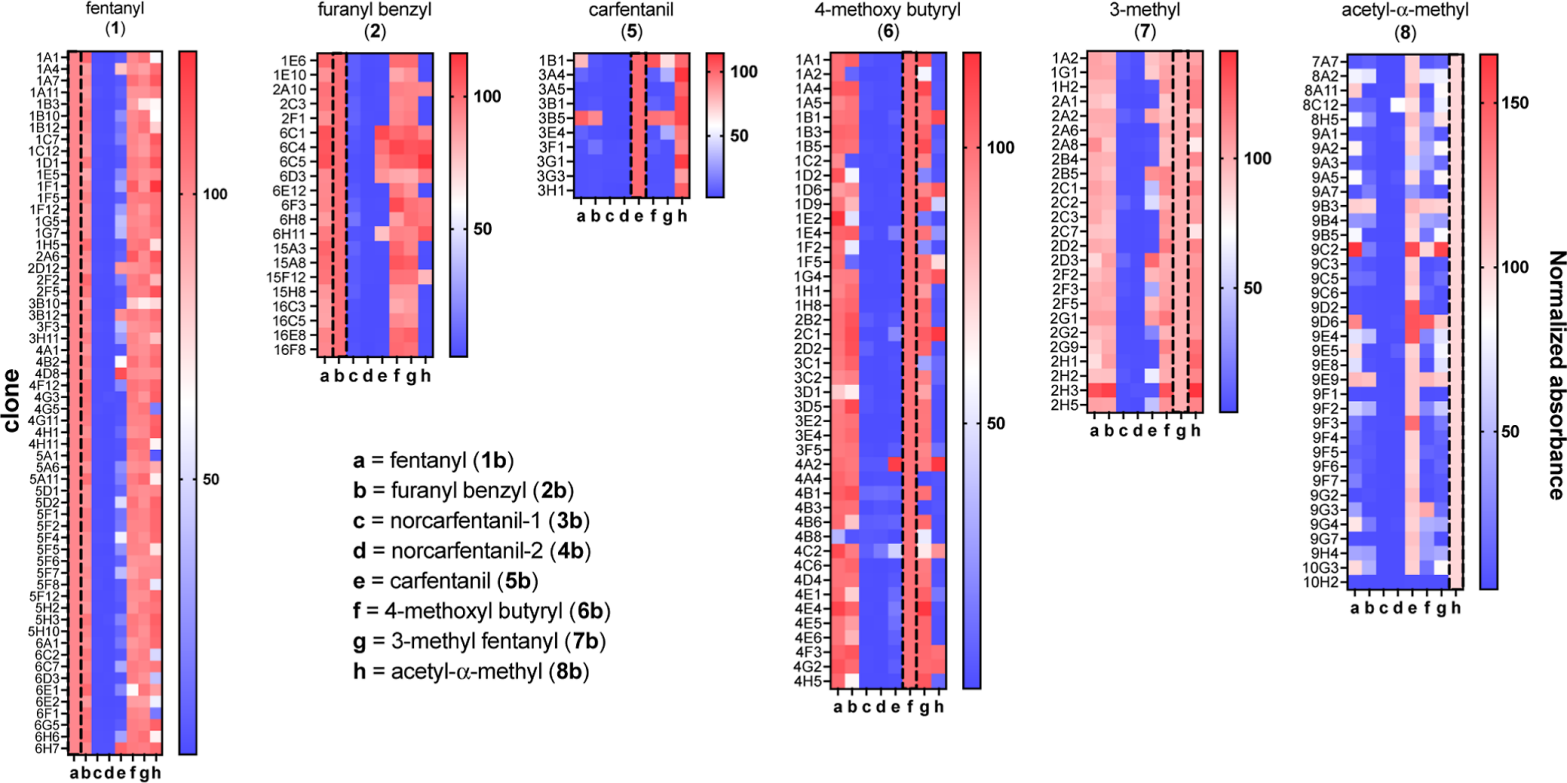
Fentanyl analogue cross-reactivity of antigen-specific clones derived from five PP7-fentanyl immunizations. Each panel is labeled at the top with the alkyne used to prepare the VLP-hapten immunogen. Supernatant (1:5 dilution in blocking buffer) from antigen-specific hybridoma clones selected from the indicated splenocytes (rows) were tested for binding to each biotinylated-fentanyl analogue (columns) adhered to a streptavidin-coated ELISA plate. Signals for each hybridoma were normalized to the value obtained for the biotinylated cognate antigen, set to 100%.

With this example of fine structural discrimination in hand, we processed the other immunizations described in Figures 2 and 3 similarly. We selected supernatants from 219 hybridoma clones derived from seven vaccinations (fentanyl, furanyl benzyl fentanyl, norcarfentanil-1, carfentanil, 4-MeO-butyryl, 3-Me, and acetyl-α-Me fentanyl) for screening by ELISA to all eight biotinylated fentanyl analogues immobilized on streptavidin-coated plates, as summarized in Figure 5. Several interesting affinity profiles emerged. As expected, the immunized antigen was usually recognized best by the antibodies derived from that immunogen. The truncated norcarfentanil metabolites (**11**, **12**) were not recognized by the vast majority of the clones tested. Clones raised against the parent fentanyl were generally highly cross-reactive to other nontruncated analogues, except for carfentanil. Conversely, carfentanil clones were the most specific to their cognate antigen. Anticarfentanil antibodies all bound acetyl-α-methyl fentanyl, and most of the larger set of antiacetyl-α-methyl fentanyl antibodies recognized carfentanil indicating a shared recognition motif or similarly immunodominant molecular feature. 3-Methyl (structures **7**/**15**) and acetyl-α-Me (structures **8**/**16**) fentanyls differ in the placement of an added methyl group and in the position of the linker, yielding a cross-reactivity pattern that again indicates the capability for finely tuned molecular recognition of small molecule antigens.

**Figure 6:**
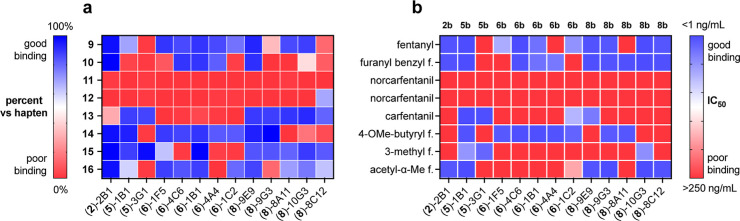
Binding profiles of purified monoclonal antibodies to immobilized and solution-phase analytes. (a) Binding of the indicated mAbs to immobilized biotinylated fentanyl derivatives listed at the left, measured by ELISA. In each case, binding of the antibody to its cognate hapten was assigned a relative value of 100%. (b) Competition ELISA of each unfunctionalized fentanyl analyte (vertical axis; “f.” = fentanyl) against the biotinylated analyte indicated across the top, which is the biotinylated cognate structure for each antibody. “good binding” means that the unmodified fentanyl analyte competed well against the biotinylated compound.

The ELISA results shown in Figure 5 for each antibody from **1**, **3**, **6**, **7**, and **8** were classified according to binding pattern and intensity. We were able to identify at least 13 unique patterns exhibited by these antibodies (Figure S12 and Table S1), some patterns shared by many antibodies and others manifested by only a single clone. From this analysis, a set of 13 clones were selected for their diverse binding patterns, listed in Figure 6. These derived from immunizations with furanyl benzyl fentanyl (**2**), carfentanil (**5**), 4-methoxy butyryl fentanyl (**6**), and acetyl-α-methyl fentanyl (**8**) conjugates, representing modifications to the acyl group, piperidine ring, the *N*-anilide ring, and the ethylbenzene alkyl connector, respectively, and including the use of both linker positions. Taken together, they cover every major structural change characteristic of the fentanyl derivatives targeted here.

Monoclonal antibodies were isolated from the selected 13 hybridoma clones and were verified to bind strongly to their cognate antigens by biolayer interferometry using the biotinylated fentanyl derivatives immobilized on a streptavidin-coated sensors. (We also included two antibodies derived from immunizations using the 3-methylfentanyl derivative **7**, although these antibodies were not used in the validation experiments below.) Table 2 summarizes these results, showing sub-nM binding avidities. We regard these numbers as indicative of very strong binding but not of discrete dissociation constants, as we cannot rule out bivalent mAb interaction with the functionalized surfaces.

**Table 2:** 1/*K*_ads_ Values for Antibody Binding to Biotinylated Cognate Haptens Determined by Biolayer Interferometry^a^

mAb	probe	1/*K*_ads_ (nM)	*R*^2^	mAb	probe	1/*K*_ads_ (nM)	*R*^2^
(**2**)-2B1	**2b**	<0.01	0.9997	(**6**)-1C2	**6b**	<0.01	0.9996
(**5**)-1B1	**5b**	0.010	0.9997	(**8**)-9E9	**8b**	0.106	0.9982
(**5**)-1B1	**8b**	<0.01	0.9984	(**8**)-9G3	**8b**	<0.01	0.9993
(**5**)-3G1	**5b**	0.025	0.999	(**8**)-8A11	**8b**	<0.01	0.998
(**6**)-1F5	**6b**	0.724	0.9993	(**8**)-10G3	**8b**	0.051	0.999
(**6**)-4C6	**6b**	0.019	0.9995	(**8**)-8C12	**8b**	0.014	0.999
(**6**)-1B1	**6b**	<0.01	0.962	(**7**)-1H2	**7b**	<0.01	0.9994
(**6**)-4A4	**6b**	0.891	0.9998	(**7**)-2G1	**7b**	0.11	0.9999

a *R*^2^ refers to the fit of a single-interaction binding isotherm to the observed data. “probe” is the molecule immobilized to the sensor surface via biotin-avidin interaction. Apparent values smaller than 1 × 10^–11^ M (0.01 nM) were judged to indicate very high affinity binding, but exact values are not reported because these experiments are likely to reflect enhancement by interactions with clusters of haptens on the surface of the sensor.

These mAbs were further examined by ELISA for recognition by our panel of eight biotinylated reagents. The results, summarized in Figure 6a, were very similar to analogous measurements using hybridoma supernatants (Figure S9), validating the use of hybridoma supernatants for screening purposes. We then employed this set of mAb reagents in the recognition of 28 fentanyl analytical standards used in our previous assessment of commercial assay kits,^18^ which were chosen because of their identification by the Drug Enforcement Administration and National Forensic Laboratory Information System as being found in multiple cases of drug seizures in the U.S.^67^Figure 6b shows the results of the assay performed similarly to that shown in Figure 5 on eight of these fentanyl derivatives, but using a range of 5 concentrations of the analyte relative to a single concentration of biotinylated competitor. This gave an estimate of the ability of the free fentanyl analogue to compete with the strong binding of the antibody to its biotinylated hapten.

Omitting the results involving norcarfentanil (for which only one positive binding event was detected), the results of the two assays matched 63% of the time (the relative degree of binding to plated antigen vs the hapten used to elicit that antibody was matched with a similar degree of binding of the corresponding fentanyl derivative in competition with the biotinylated hapten). However, in 19 out of 78 cases, strong binding to the plated biotinylated antigen did not correlate with effective recognition of the nonbiotinylated analogue, and in 10 cases, weak binding of the plated antigen (relevant to the immunogen structure) was paired with good recognition of the free nonbiotinylated analyte. No trend could be discerned that would implicate the position or nature of the linkers used to attach biotin to the structures, but these results serve as a reminder that binding to immobilized small molecules may indeed differ from recognition of the structures in solution.

**Figure 7:**
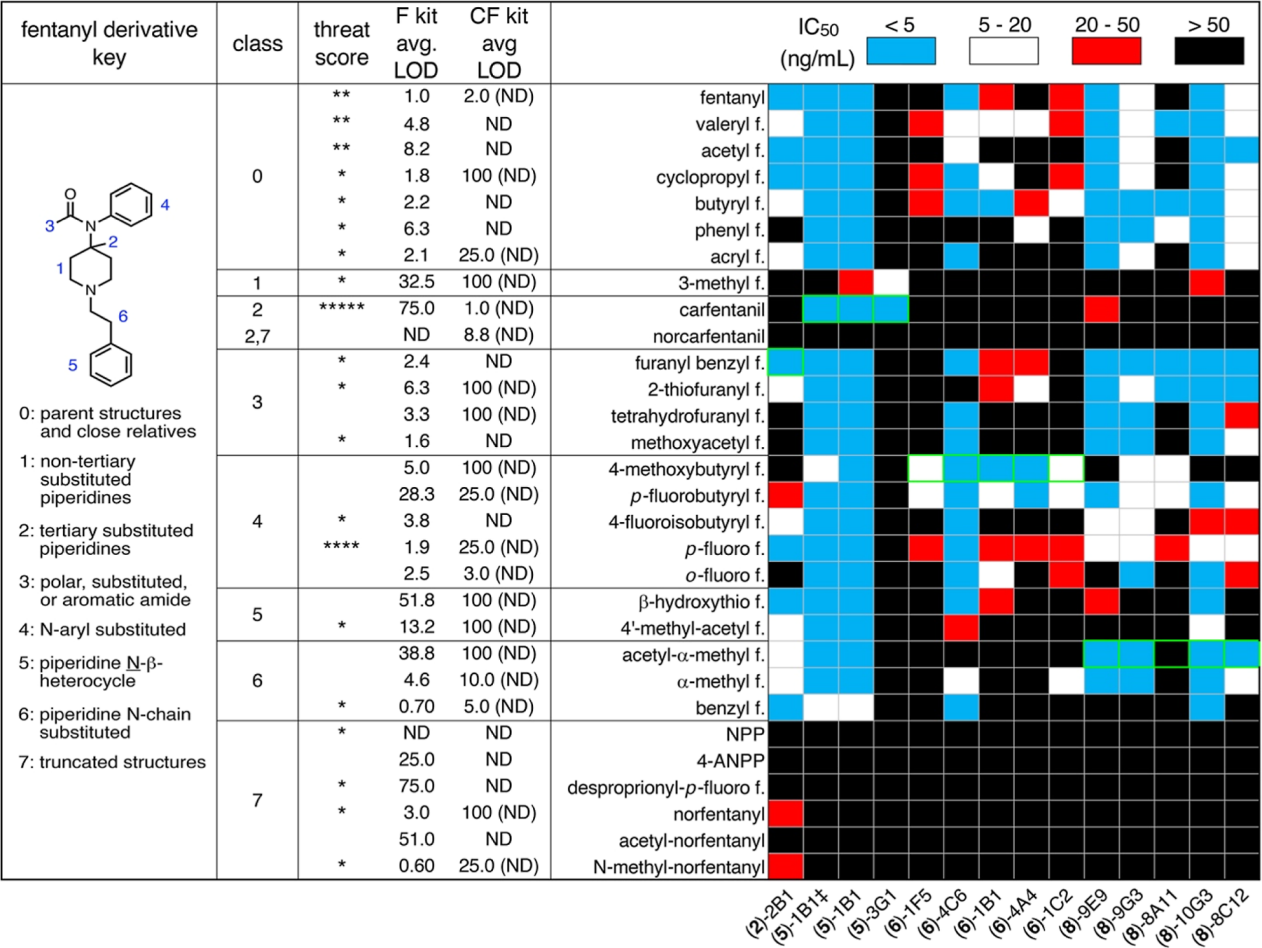
Binding of monoclonal antibodies to fentanyl derivatives. Estimated IC_50_ values from competition ELISA performed as shown in Figure 5 with five concentrations of the indicated fentanyl derivative (0.008–25 ng/mL) in the presence of 0.5 ng/mL of the biotinylated hapten structure used to elicit the antibody, except for (**5**)-1B1‡, which was performed with biotinylated acetyl-α-methyl fentanyl (**8b**). Entries boxed in green denote analysis involving the fentanyl derivative corresponding to the hapten used to generate the indicated antibody. Values of >50 indicate the observation of very weak or no competition. “Threat score” denotes how often the indicated fentanyl appeared on DEA and CFSRE publications from 2019 to 2021. Each asterisk denotes 1–2 appearances on these lists. “F kit avg. LOD” denotes the average limit of detection of commercial antifentanyl kit(s) to detect the indicated analyte as reported in ref (18) “CF kit avg LOD” has the same meaning for commercial kits against carfentanil. ND indicates no detection of the indicated compound; the appearance of numerical value appears with (ND) denotes that some of the kits used did not detect the indicated compound at all in repeated tests.

Figure 7 shows the full set of results for the use of the 13 selected antibodies against the full panel of 28 fentanyl derivatives. Observations of interest include the following.

(i) Except for the aforementioned truncated (Class 7^18^) analytes, all of the fentanyl derivatives could be detected by multiple mAbs, and many antibodies were able to recognize most of the derivatives. High-affinity detection of all full-sized fentanyl derivatives in Figure 7 (except norcarfentanil) is possible with only two antibodies. However, some were quite selective, allowing for the pairwise discrimination between all of the fentanyl derivatives shown by the selection of 2–3 mAbs in each case, and for the identification of each derivative from all the others using a panel of 4–5 mAbs. Furthermore, for most derivatives, the observed IC_50_ in our competition ELISA experiments was significantly smaller than the average limit of detection of the commercial kits.
(ii) One of the anticarfentanil antibodies had broad specificity [(**5**)-1B1] and the other was exquisitely selective for only carfentanil and 3-methylfentanyl [(**5**)-3G1]. Both of the latter compounds have an extra substituent constituting a steric branch at or near the piperidine 4-position.
(iii) Two mAbs [(**6**)-4C6 and (**6**)-1C2] apparently recognize α-methyl fentanyl significantly better than they recognize the very closely related acetyl-α-methyl fentanyl, suggesting that the additional methyl group (propionyl vs acetyl amide) is part of the binding motif. Conversely, no significant difference was observed in antibody recognition of *p*-fluorofentanyl and *p*-fluorobutyryl fentanyl (the latter also having an extra methyl group on the amide carbonyl component), but recognition of the branched *p*-fluoroisobutyryl fentanyl was lost by four mAbs.
(iv) Two Class 3 analytes, 2-thiofuranyl and tetrahydrofuranyl fentanyls, differ only in the nature of the heterocyclic amide carbonyl substituent. One antibody [(**6**)-4C6] discriminates completely in favor of the unsaturated furan while three mAbs [(**6**)-1B1, (**6**)-4A4, and (**8**)-8A11] bind the thiofuran compound much better. The recognition pattern of methoxyacetyl fentanyl is the same as the tetrahydrofuran derivative, justifying their inclusion in the same class.
(v) Only two *N*-benzyl structures were included (benzylfentanyl and furanyl benzylfentanyl), which were recognized very similarly by mAbs raised against **2** and **5**, but discriminated by mAbs produced by immunization with **6** and **8** (both sets of which largely recognized the furanyl benzyl structure but not benzylfentanyl). This highlights the fact that details of hapten recognition cannot be reliably predicted, as neither **6** nor **8** contain a benzyl moiety and they have the linker attached at different ends of the structure.
(vi) Antibody (**8**)-8A11 shows an unusual dichotomy of binding behavior: it strongly binds its eliciting hapten when biotinylated and immobilized on an interferometry sensor (Table 2), but does not apparently recognize the free drug (acetyl α-Me fentanyl) in a competition ELISA experiment against the plated biotinylated molecule. It also fails to recognize other fentanyl derivatives that are bound by a number of other mAbs generated by immunization with the VLP conjugate of **8.** This suggests that (**8**)-8A11 recognizes a motif that includes a portion of the linker that is present on the biotinylated molecule but not the drug.

The sequences of the variable regions of the mAbs in Figure 7 fall into several distinct groups (color coded in Table S2). The CDR regions of three antibodies among our set closely match certain corresponding sequences of antifentanyl antibody HY6-F9 derived from hapten immunization, recently structurally characterized by Pancera and colleagues^55^ (Table S3). We can therefore assume that (**6**)-4C6, which shows similar hapten affinity as HY6-F9, binds in a similar manner, enclosing the piperidine ring in a pocket with hydrogen bonding to its tertiary nitrogen and engaging the rest of the molecule over a large surface area.^55^ Molecular modeling of fentanyl binding by other high-affinity antibodies by Matyas and co-workers proposes other binding modes including π–π and cation–π interactions.^68^ Notably, our three mAbs that have sequences related to HY6-F9 exhibit very different patterns of fentanyl analogue recognition (Figure 7 and Table S3). High-affinity antibody binding to small motifs typically requires induced-fit maturation by somatic mutation, and the results have often been characterized by limited antibody diversity.^69,70^ These reported antibody-fentanyl structural results, along with our observations of different groups of CDR sequences and binding patterns in our high-affinity mAb clones, support the idea that a variety of intermolecular interactions are employed in the affinity maturation process.

## Conclusions

The trends described above are largely reconciled when considering the position of the hapten linker, which was chosen to place the key structural feature of each derivative as far from the linkage location as possible. The results of Figure 5 show distinct properties for the structures bearing alkyne linkers at the aniline-amide “head” position (norcarfentanils, carfentanil, acetyl-α-methyl fentanyl) vs those with the linker at the ethylbenzene “tail” position (fentanyl, furanyl benzyl, 4-methoxy, and 3-methyl fentanyls). For example, more clones favoring 3-methyl (**7**, connected at the tail) over acetyl-α-Me (**8**, connected at the head) fentanyl were produced by VLP conjugates to tail-connected haptens [furanyl benzyl (**2**), 4-methoxy butyryl (**6**), and (to a lesser extent) the parent fentanyl (**1**)]. In contrast, vaccines based on carfentanil (**5**) and acetyl-α-Me fentanyl (**8**), connected at the head, gave more antibodies favoring acetyl-α-Me over 3-methyl fentanyl. Janda and colleagues have recently described exciting progress in the creation of vaccines against fentanyl variants. These advances include a KLH conjugate of a fentanyl derivative class not explored here (having a benzimidazole group in place of the ethylbenzene aromatic ring)^71^ and tetanus toxoid conjugates of carfentanil differing in the point of attachment as in our approach above.^36^ In the latter case, cross-reactivity with a more limited set of eight fentanyl analogues showed similar dependence on the linker position.

In addition to their value as diagnostic or detection reagents, the use of mAbs as therapeutic agents is receiving increasing attention.^39,68,72^ While the ability of antibodies to bind different fentanyl derivatives has been reported previously,^31,68,73^ the work presented here constitutes the most extensive exploration of this topic so far in terms of the number and diversity of immunogens employed and derivatives tested. We show here that analytical discrimination between closely related fentanyl derivatives, as well as detection of a broad range of such derivatives, is possible with a limited set of antibodies. While this may show that it is challenging to create “broadly neutralizing” therapeutic or prophylactic antibodies against current and emerging structural variations of fentanyl, the field has not yet fully harnessed the immune system to meet this challenge.

## Methods/Experimental

### Vaccine Design and Characterization

Wild-type PP7 nanoparticles and PP7-fentanyl conjugate vaccines were produced as previously described.^1,2^ VLPs were modified to display azides by mixing NHS-ester azido-acetate linkers in DMSO and phosphate buffer in molar ratios of 1:2–4 or 1:10 lysine: NHS-N_3_ overnight at 4 °C. Alkyne-labeled fentanyl derivatives were installed by Copper catalyzed azide–alkyne cycloaddition (CuAAC) in 1:0.25 molar ratios VLP-N_3_: Alkyne-fentanyl in the presence of ligand, CuSO_4_, aminoguanidine, sodium ascorbate and DMSO for 90 min at 50 °C. Conjugates were purified by PD-10 column (GE) chromatography, followed by concentration via centrifugation through 100,000 Da molecular weight cutoff filter (Millipore Sigma). Protein concentrations were determined by Bradford Assay using Coomassie Plus Protein reagent (Pierce) with bovine albumin standard. At each step, VLP-conjugates were analyzed for size, diameter, and monodispersity by Dynamic Light Scattering (DynaPro, Wyatt Technology), and purity, aggregation and particle stability after modification determined by size-exclusion chromatography (Superose 6). Linker and antigen density on VLPs were measured by liquid-chromatography time-of-flight mass spectrometry (Agilent).

### Immunizations

Six-week-old female pathogen-free BALB/c mice were obtained from Charles River Laboratories and were immunized subcutaneously on both sides of lower anterior abdomen on day 0 followed by boost inoculations. Details are provided in Supporting Information. Body mass was measured over time as a criterion of general health and vaccine safety and did not vary from particle-treated control mice. Mice selected for high antifentanyl conjugate serum antibody levels were sacrificed, followed by splenectomy, B cell extraction, hybridoma creation, and analysis by ELISA and biolayer interferometry as described in Supporting Information.

### Fentanyl Analogue Competition Binding (Figures 5 and 7)

These experiments were performed by similar procedures (Supporting Information) with the following differences: Figure 5 employed hybridoma supernatants and competition between each of 29 unmodified fentanyl derivatives (purchased from Cayman Chemicals) mixed with **10** (50:1 molar ratio of the fentanyl derivative); Figure 7 employed purified monoclonal antibodies and competition between each unmodified fentanyl derivative and its cognate biotinylated antigen.

## Supporting Information Available

Detailed experimental methods describing vaccine design and construction, characterization, animal studies, and antibody isolation and characterization (PDF)
