# Supplementary material for: Substructure-Specific Antibodies Against Fentanyl Derivatives
Source: ACS Nano. 2025 Jan 10;19(3):3714–25. doi: 10.1021/acsnano.4c14369 (PMC11781026; doi:10.1021/acsnano.4c14369)
Supplement: Supplementary file 1 — nn4c14369_si_001.pdf [file nn4c14369_si_001.pdf]

Supporting Information

Substructure-Specific Antibodies Against Fentanyl Derivatives

Asheley P. Chapman,[1,‡] Minghao Xu,[1,‡] Michelle Schroeder,[1,‡] Jason Goldstein,[2] Asiya Chida,[2] Joo R. Lee,[2] Xiaoling Tang,[2] Rebekah Wharton,[3]* M.G. Finn,[1,4]*

[1] School of Chemistry and Biochemistry, Georgia Institute of Technology, 901 Atlantic Dr., Atlanta, GA 30332, USA

[2] Immunodiagnostic Development Team, Preparedness, Response, & Outbreak Services Branch, Division of Core Laboratory Services & Response, Office of Laboratory Systems and Response, Centers for Disease Control and Prevention, 1600 Clifton Rd NE., Atlanta, GA 30333, USA

[3] Division of Laboratory Sciences, National Center for Environmental Health, Centers for Disease Control and Prevention, 4770 Buford Hwy, Atlanta, GA 30341, USA

[4] School of Biological Sciences, Georgia Institute of Technology, 901 Atlantic Dr. Atlanta, GA 30332, USA

Table of Contents

METHODS 2
- *Animal studies* 2
- *Hapten dose calculation* 2
- *Immunizations and B cell extraction* 2
- *Hybridoma fusion* 2
- *Development of anti-fentanyl mAbs* 3
- *ELISA* 3
- *Monclonal antibody binding by biolayer interferometry (BLI)* 4
- *Fentanyl analogue competition ELISA (Figure 5)* 4
- *Fentanyl analogue competition ELISA (Figure 7)* 4

PARTICLE CHARACTERIZATION 5

EXPLORATION OF ADJUVANTS 8

SAFETY AND SPECIFICITY 9
- *Non-toxicity* 9
- *IgM response* 9
- *Non-reactivity with naloxone* 10

ANTIBODY BINDING PATTERNS FROM IMMUNIZATION WITH FURANYL BENZYL FENTANYL HAPTEN (2) 10
- *Fentanyl derivative structure classifications* 12
- *Hybridoma selection of fentanyl- vs. PP7-specific clones* 13

SCREENING OF HYBRIDOMA SUPERNATANTS 14
- *Supernatants vs. purified antibodies* 14

PATTERNS OF FENTANYL ANALOGUE RECOGNITION 15

SCREENING OF 180 SELECTED CLONES 16

SEQUENCES OF SELECTED CLONES 22

CHEMICAL SYNTHESIS AND CHARACTERIZATION 23

REFERENCES 24

METHODS

Animal studies. All animal studies were performed in compliance with the Georgia Institute of Technology Institutional Animal Care and Use Committee (protocol A-170063) and all protocols followed National Institute of Health Guide for the Care and Use of Laboratory Animals guidelines. Mice were housed in the Physiological Research Laboratory at Georgia Tech.

Hapten dose calculation. Hapten doses were calculated based on number of haptens per VLP (Fig. 2) and the molecular weight of the hapten, as follows.

- The PP7 VLP has a molar mass of 2,497,320 (180 subunits, each 13.874 kDa).
- The fractional mass of each hapten delivered per mass of VLP is given by:

(MW hapten) x (# haptens per VLP) / (MW VLP)

For example, for the fentanyl alkyne (**1**, average of 228 haptens per VLP), every gram of VLP carries 0.038 g of hapten. Thus, 50 µg of VLP bears 1.9 µg of this hapten. Note that particle concentration is determined by Bradford assay, which does not detect the hapten.

Immunizations and B cell extraction. Six-week-old female pathogen-free BALB/c mice were obtained from Charles River Laboratories (Wilmington, MA). Mice were immunized subcutaneously on both sides of lower anterior abdomen on day 0 followed by boost inoculations at day 14 (week 2) and day 28 (week 4) with 50 ug of total VLP per dose in 0.1 mL volume in 0.1 M sterile potassium phosphate buffer; mice selected for hybridoma generation were boosted 3 days prior to harvest with 10 ug of VLP-conjugate in 0.1 mL. Mice in adjuvant screen were immunized subcutaneously on day 0 with 50 µg in 100 uL VLP-**5** conjugate in 0.1M KPO_4 (no adjuvant group), with 500 ng PBS-57, an α-galactosylceramide derivative specific for NKT cell activation (and thus designated "NKT"),[3] co-mixed in DMSO immediately prior to injection (NKT group), or 1:1 with Titermax Gold oil-in-water emulsion (Titermax group). The no-adjuvant group was boosted on days 14 and 28, NKT group on day 42, and Titermax group on day 42 with no additional adjuvant. Blood was collected by submandibular bleed at days 0 (prior to immunization), 14, 21, 28, 35 and 43 (terminal). Body mass was measured over time as a criterion of general health and vaccine safety and did not vary from particle-treated control mice. Mice selected for high anti-fentanyl conjugate serum antibody levels were sacrificed by CO_2 asphyxiation on day 43 (Fentanyl, Norcarfentanil-1, Acetyl-α-Me-Fen, 3Me-Fen), day 50 (Carfentanil and Norcarfentanil-2), or day 35 (CF1, 4-OMe-Butyryl) followed by splenectomy and B cell extraction. These timepoints were dictated by personnel scheduling restrictions during the early stages of the global SARS-CoV-2 pandemic when these experiments were performed, but serum antibody titers were very similar throughout the day 35-53 period.

Hybridoma fusion. The step-by-step protocol was as follows.

Spleen preparation and B cell harvesting

- Transfer spleen to sterile dish, and rinse the exterior with PBS, removing any remaining debris and/or extraneous fibrous tissue. Transfer the sample to a second sterile dish.
- Inject spleen with IMDM in ≈0.5 mL aliquots using a 23-gauge needle; total volume = 20 mL. This is done by holding the spleen with forceps and injecting in various locations with media. The injection process should fill the spleen with enough medium that B cells are extracted out of the spleen and into the dish with each injection. Spleen will begin to look "empty" after successful extraction.
- Transfer the resulting cell suspension extracted from spleen to a 50 mL tube.
- Pellet splenocytes (and resuspended myeloma in parallel) by centrifugation at 400 x g (1569 RPM) for 7 minutes. Pour off the supernatant.
- Resuspend B cells in 5 mL 1X lysis buffer and incubate on ice for 5 minutes with shaking every 60 seconds. This process will lyse the red blood cells in the cell suspension.
- Quench the lysis reaction by adding 25 mL 1x PBS and centrifuge at 400 x g (1569 RPM) for 7 minutes.

- Aspirate the supernatant, resuspend B cells in 20 mL IMDM, and count (Beckman cell counter). Medium should be free of serum, as serum can inhibit the fusion process.

Mixing and Pre-electrofusion

- SP2-IL6 myelomas (ATCC) cells were cultured in IMDM supplemented with 10% FBS (Gibco).
- Mix cells (B cells and myelomas) at 1:1 ratio in 45 mL IMDM (serum-free).
- Centrifuge at 400 x g (1569 RPM) for 7 minutes.
- Pour off supernatant and resuspend cells in 2 mL IMDM (serum-free). Add 50 μL pronase (10 mg/mL) and incubate at RT for 3 minutes. In this reaction, the total cell number varied between 5 x 10^7 and 2 x 10^8. Quench this reaction by adding 0.5 mL low-IgG FBS.
- Add 48mL IMDM (serum-free) and centrifuge at 400 x g (1569 RPM) for 7 minutes.
- If performing multiple electrofusion reactions, split reactions into 50 mL tubes and keep in IMDM.

Electrofusion

- Resuspend cells in 20 mL BTX Cytofusion media (cold). Centrifuge at 400 x g (1569 RPM) for 7 minutes.
- If performing multiple reactions, only add cytofusion media to 2 tubes at a time. *Note: Keeping cells in cytofusion media for longer than 30 minutes will alster the electrophysiological properties of the cells and can lead to inefficient fusion.*
- Repeat the suspension in cold BTX Cytofusion media and centrifugation a second time.
- Resuspend cells in 10 mL Cytofusion media, and add this cell suspension to the chamber of a BTX ECM 2001 Electro Cell Manipulation Pulse Generator with 630B Safety Stand (Harvard Apparatus). Electrofusion under automatic mode was performed for 5 minutes at a field strength of 3000 V/cm. Perform the fusion within 10 minutes after the final wash.
- The cells were then mixed with 40 mL pre-warmed HAT (hypoxanthine-aminopterin-thymidine) medium containing aminopterin and incubated in a 37°C water bath for 30 minutes before transferring to a T75 flask and cultured at 37°C with 5% CO_2.

Immortal B-cells (HGPRT negative) cannot synthesize nucleotides by the salvage pathway, and *de novo* nucleotide synthesis is blocked by aminopterin. Thus, the only cells to remain are hybrids formed by the fusion of antibody-producing and immortal B cells, since functional HGPRT activity (nucleotide synthesis by the salvage pathway) is provided by the antibody producing B cell partner.

Development of anti-fentanyl mAbs**.** Fused hybridoma cells were plated into semi-solid methylcellulose-based media containing hypoxanthine, aminopterin, thymidine, and CloneDetect reagent. Automated selection using a ClonePix II instrument (Molecular Devices) isolated IgG-positive colonies. To determine antigen specificity, hybridoma supernatant from confluent wells were diluted 1:5 in blocking buffer and screened against 0.25 μg/mL biotin-fentanyl by indirect ELISA (each conjugate was screening against its cognate biotinylated derivate). Most mAbs were tested in duplicate and positive reactivity was scored if the average OD value was greater than 2.0 in order to select for strongest binders. This stringent criterion was necessary to choose among many hapten-binding clones. IgG-producing clones were cultured in static T-75s in IMDM (Gibco) /10% Low IgG FBS at 5% CO_2/37°C. mAbs were purified by Protein G Sepharose Fast Flow and eluted at >0.2 mg/mL in 100 mM glycine/150 mM NaCl (pH 2.8) and neutralized with 200 mM Tris (pH 8.0). Antibodies were isotyped using strips with anti-mouse heavy chain capture antibody (anti-IgG1, IgG2a, IgGb, IgG3, IgA and IgM) or anti-mouse light chain (kappa or lambda) and analyzed by SDS-PAGE and Superdex-200 size-exclusion chromatography. Aliquots of mAbs were stored at 4°C until use. Protein concentrations were determined by NanoDrop 2000c spectrophotometer at 280 nm.

ELISA**.** Anti-fentanyl immune responses, antigen specificity of immunized mouse sera, and antigen recognition of hybridoma supernatants were analyzed by ELISA. Streptavidin was plated on half-area high-binding polystyrene plates (Corning) overnight at 1 ug/mL at 4°C in PBS. Unbound protein was washed away with PBST (1x PBS with 0.5% Tween) followed by blocking with casein buffer (PBS with 1% casein,

w/v; VWR International) for 1 h at room temperature with mild shaking (55 rpm). For antigen-specificity, cognate biotin-fentanyls were plated at 0.5 μg/mL or 0.25 μg/mL in blocking buffer 1 h at room temperature; for antigen cross-reactivity, the same concentration of all other biotin-fentanyls were plated (0.25 μg/mL). Plates were again washed with PBST and serum or supernatant samples were then incubated for 1 h at room temperature with shaking, followed by washing with PBST. For serum titers, six dilutions of sera in blocking buffer were plated; for supernatant specificity and antigen cross-reactivity experiments, hybridoma supernatant (1:4 [all others] or 1:10 for parent fentanyl) was plated. Secondary reporter goat anti-mouse IgG HRP (Southern Biotech) was diluted (1:2500 serum, 1:3000 supernatants) or goat anti-mouse IgM HRP in blocking buffer and incubated for 1 h at room temperature with shaking, followed by washing. Plates were developed by adding 1-step Ultra TMB (Fisher Scientific) for 30 s, followed by quenching with 2M H_2SO_4. Absorbance (450 nm) was measured by plate reader (Varioskan Flash, Thermo Fisher Scientific). Titers were calculated by sigmoidal non-linear regression using GraphPad Prism v.8 analysis with log 10 serum dilution plotted against absorbance at 450 nm.

Monclonal antibody binding by biolayer interferometry (BLI)**.** Binding of mAbs to their respective biotinylated fentanyls were characterized by BLI on an Octet Red 96 (ForteBio) at 30°C with agitation at 1000rpm. Prior to analysis, streptavidin (SA) biosensors were hydrated in kinetics buffer (PBS, 0.05% bovine serum albumin, 0.02% Tween20) for 10 min at room temperature. For analysis, baseline was collected by dipping sensors in kinetics buffer for 60s. Sensors were then loaded with 0.125 µg/mL of biotinylated fentanyl in kinetics buffer for 300s. Baseline was collected again by dipping sensors in kinetics buffer for 60s. MAbs (1.25 – 5 µg/mL in kinetics buffer) were then associated for 300s followed by dissociation for 300s in kinetics buffer. Equilibrium dissociation constants (K_d) were calculated in the Octet Data Analysis 9.0 software using a global 1:1 Langmuir model.

Fentanyl analogue competition ELISA (Figure 5). High-binding plates were purchased pre-coated with Protein G and washed with PBST buffer (3x). Hybridoma supernatants were diluted to 1 μg/mL in Complete Medium and 100 μL/well was added and incubated 1h at RT with gentle shaking (55 rpm). In a separate plate, 29 unmodified fentanyl compounds (purchased from Cayman Chemicals) were diluted to 0.2 μg/mL in casein buffer and mixed 1:1 (vol/vol) with a solution of **10** (4 ng/mL), establishing a 50-fold excess of the fentanyl derivative. [The solution of **10** was prepared by diliution of 1 mg/mL stock in DMSO with PBS buffer (1:1000), followed by dilution in casein buffer (1:250)]. Plates were again washed with PBS (3x), followed by addition of 100 uL of each mixture of fentanyl analog and **10** in triplicate. After incubation for 1h at room temperature with gentle shaking (55 rpm), the plates were washed (3x) and each well was treated with 100μL of 1:20,000 streptavidin-HRP (ThermoFisher) diluted in PBS + 1% BSA. After incubation for 1 h at room temperature with gentle shaking, each plate was washed (3x) and each well was treated with 100 μL of TMB substrate, allowed to develop for 15-30 min, quenched by addition of 100 μL/well of TMB stop solution, and read as absorbance at 450 nm. on a microplate reader.

Fentanyl analogue competition ELISA (Figure 7). This was performed with a method slightly modified from the original procedure above. High binding 96-well half area microplates (Corning) were coated overnight at 4°C with 100 µL of 1 µg/mL mAb in carbonate buffer. Wells were washed three times with 150 µL PBST and then blocked for 1 hour at room temperature with 150 µL Blocker BSA (ThermoFisher). 100 µL of unlabeled fentanyl analog (Cayman Chemical) (0.008-25 ng/mL) pre-mixed with 0.5 µg/mL cognate biotinylated fentanyl in Blocker BSA were added to the ELISA plates and incubated for 1 hour at room temperature. Plates were then washed with 150 µL PBST. Streptavidin-HRP (100 µL, ThermoFisher) diluted 1:25,000 in blocker BSA was then added to each well and incubated at room temperature for 1 hour. Wells were washed three times in PBST, followed by addition of 1-step Ultra TMB ELISA substrate solution (100 µL, ThermoFisher) to each well, incubation for 5 min at room temperature, and addition of 50 µL of 0.16M sulfuric acid stop solution. Color change was read at 450 nm on a microplate reader.

Particle characterization

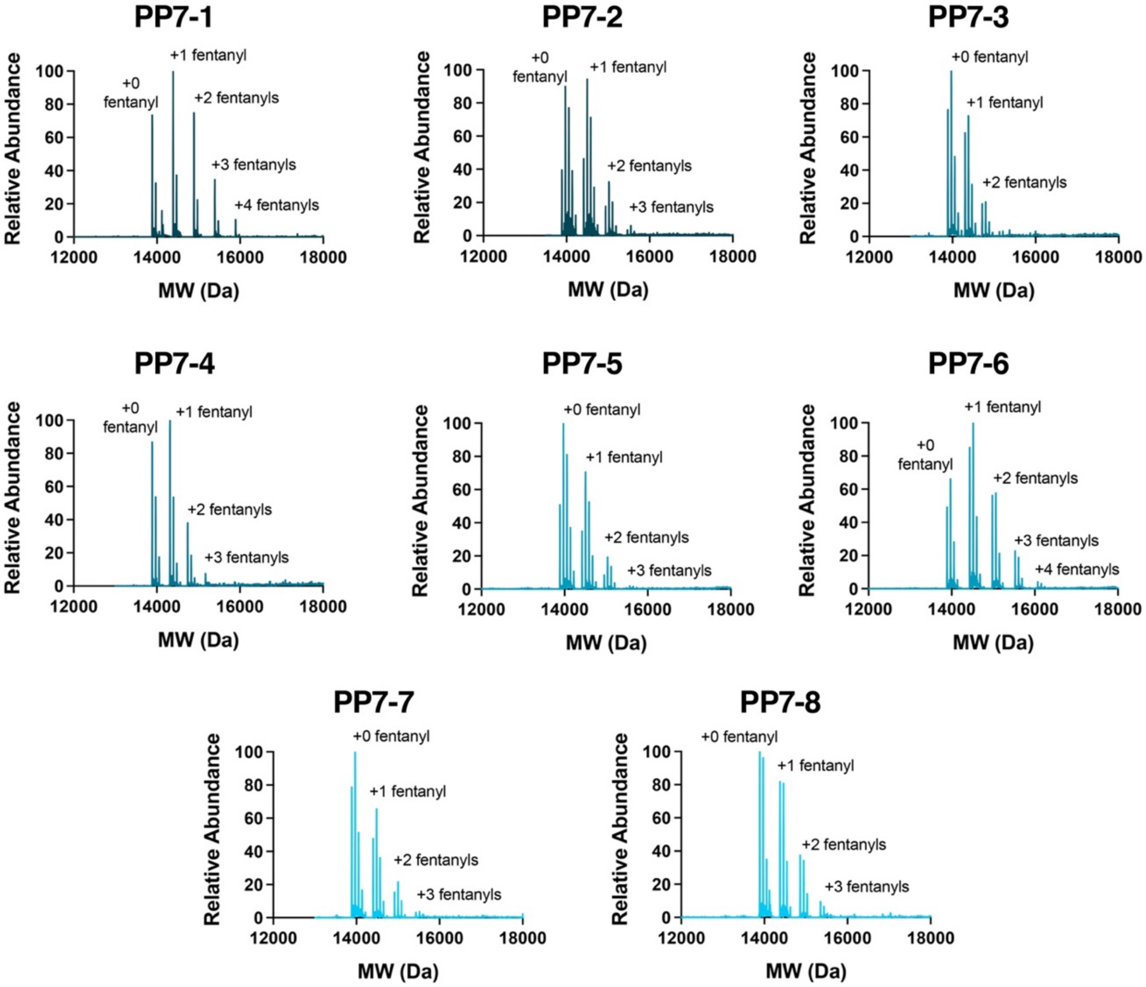

Figure *S*1. Mass spectrometric analysis of VLP-fentanyl conjugate vaccines. Azide-linker and alkyne-fentanyl antigen density per coat protein of PP7 (unmodified mass: 13890 Da) were determined by HPLC (C3 reversed phase column) followed by time-of-flight mass spectrometry (electrospray ionization).

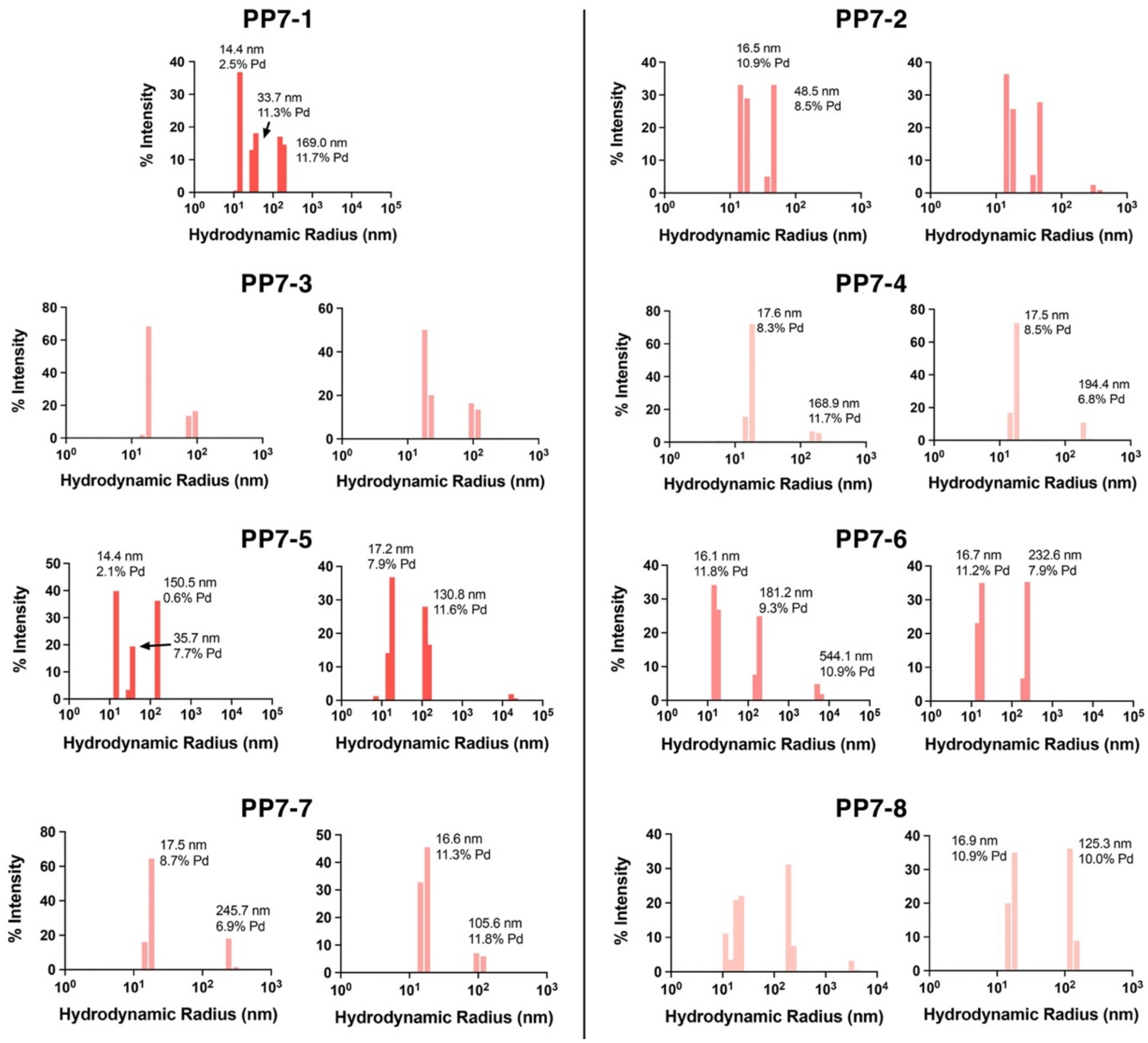

Figure S2. Dynamic light scattering analysis of VLP-fentanyl conjugates.

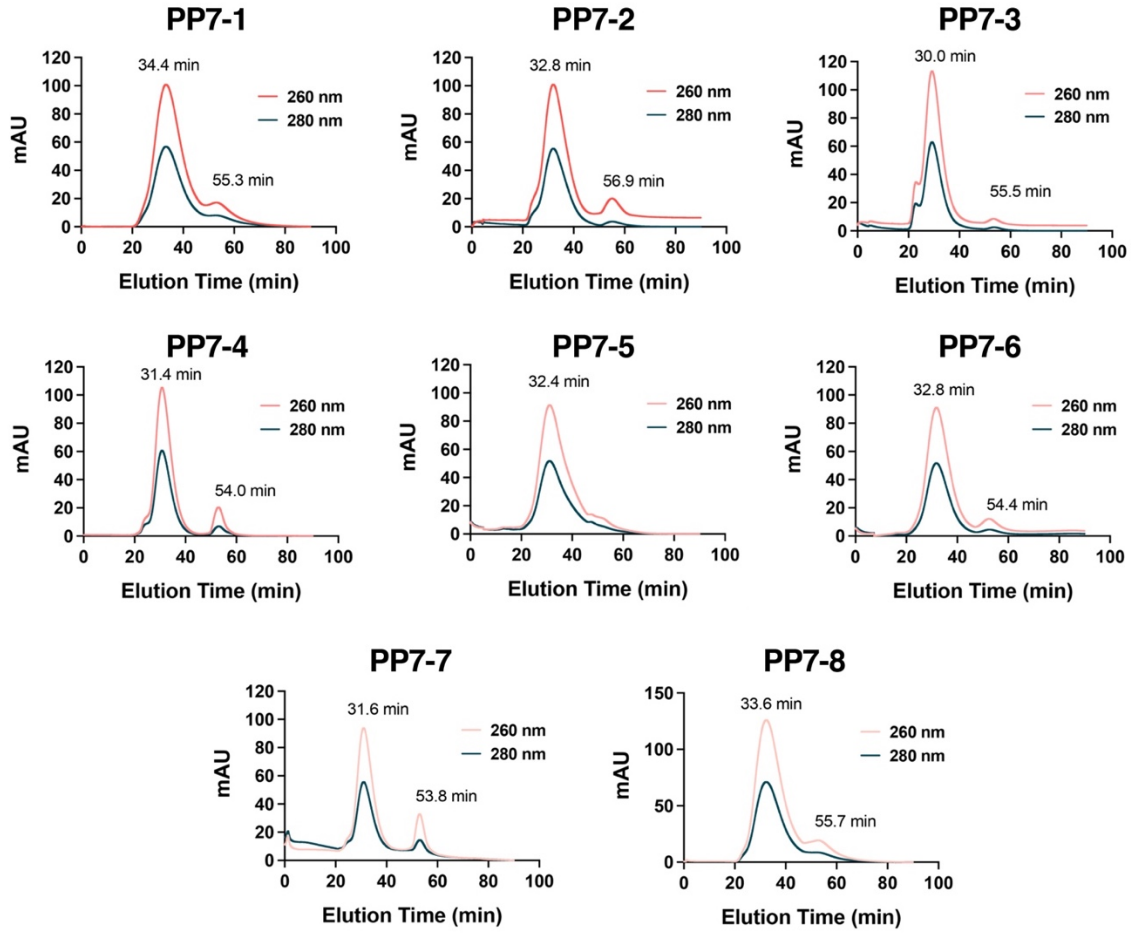

Figure S3. Fast protein liquid chromatographic analysis of VLP-fentanyl conjugate vaccines. Purified VLP-fentanyl reactions were analyzed for vaccine purity and to assess degree of aggregation on Sephadex column chromatography. Protein and encapsulated mRNA monitored at 260 and 280 nm, respectively. Major peak at ~30 min represents monomeric VLP-conjugate, secondary peak indicates aggregation.

Exploration of adjuvants

We tested two adjuvants with the **PP7-2** immunogen: the commercially-available water-in-oil emulsion TiterMax Gold® (designated "TMX"),[5] and PBS-57, an a-galactosylceramide derivative specific for NKT cell activation (and thus designated "NKT"),[3] with results summarized in Figure S4. Secondary boosts were delayed to week 8 to allow time for immune cell recovery (Fig. S4a). Similar endpoint titers were observed, but the NKT adjuvant elicited a sharp enhancement of titer in two of the three animals tested (Fig. S4b). Mice given no adjuvant or TiterMax in the prime immunization produced more IgG_{2a}, commonly associated with a pro-inflammatory Th-1 response,[6] than mice given NKT adjuvant in both prime and boost inoculations (Fig. S4d); the NKT-treated mice produced less IgM against the hapten (Fig. S4c). Comparing titer profiles at week 9 (after which all groups had received at least one boost), formulations were not significantly different, leading us to select a single mouse from each group (mouse with highest anti-**2-b** titer at week 12) for hybridoma generation.

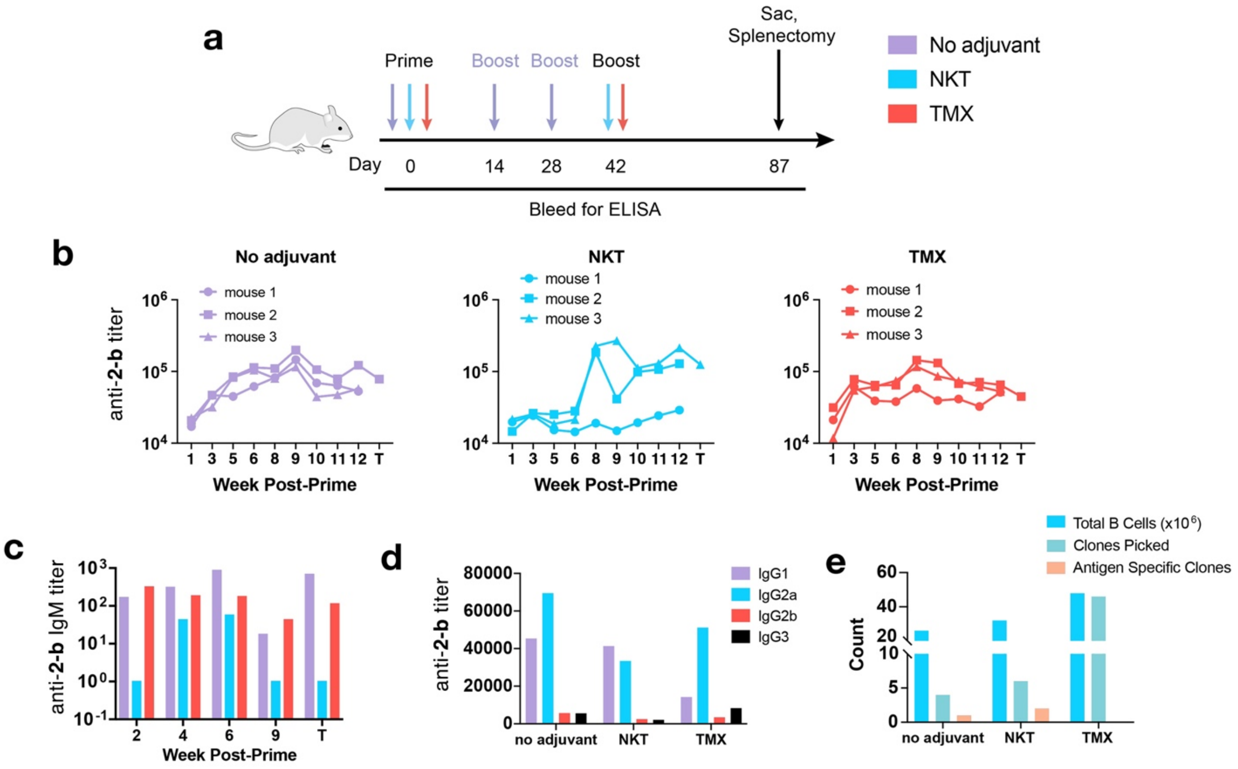

Figure S4. Adjuvant optimization of PP7-furanyl benzyl fentanyl (PP7-2) vaccines. a) Vaccine schedule of PP7-Furanyl benzyl fentanyl adjuvant screen in BALB/c mice. 50 µg VLP-conjugate was injected on days 0, 14, and 28 (no adjuvant), days 0 and 42 (with NKT adjuvant), and days 0 and 42 (TMX included in prime only). b) IgG response against plated fentanyl derivative **2-b**, as measured by ELISA. Points represent mean and error bars are SEM (n= 3 per group). c) Anti-fentanyl 10 IgM response over time measured by ELISA. Pooled mouse sera, weeks 2, 4, 5, 9 and 13 post-prime. d) IgG subclass distribution per vaccine adjuvant screened measured by ELISA. Pooled mouse sera, week 9 post-prime (n = 3 mice per group). e) Hybridoma statistics of mice selected for sacrifice at week 13 post-prime (n = 1 mouse per group). Clones picked based on total IgG secretion measured by anti-IgG FITC, selected by ClonePix; antigen specific clones determined by anti-**2-b** ELISA. T = terminal.

The TMX mouse resulted in the highest number of viable B cells from spleen perfusion and in 46 IgG-secreting clones picked compared to only 4 or 6 for non-adjuvanted or NKT mice, respectively. However, when supernatant from these clones was screened for binding to biotinylated hapten **2-b**, none of the TMX clones were antigen specific, whereas 25-30% of the few IgG-secreters from non- adjuvanted or NKT clones bound cognate antigen (Fig. S4e). Obviously, such a small sample size (n= 3 in vaccinations, n=1

in the hybridoma groups), is not large enough to draw overarching conclusions, but taking these results into account, we chose to employ the NKT adjuvant in primary immunizations.

Safety and specificity

Non-toxicity

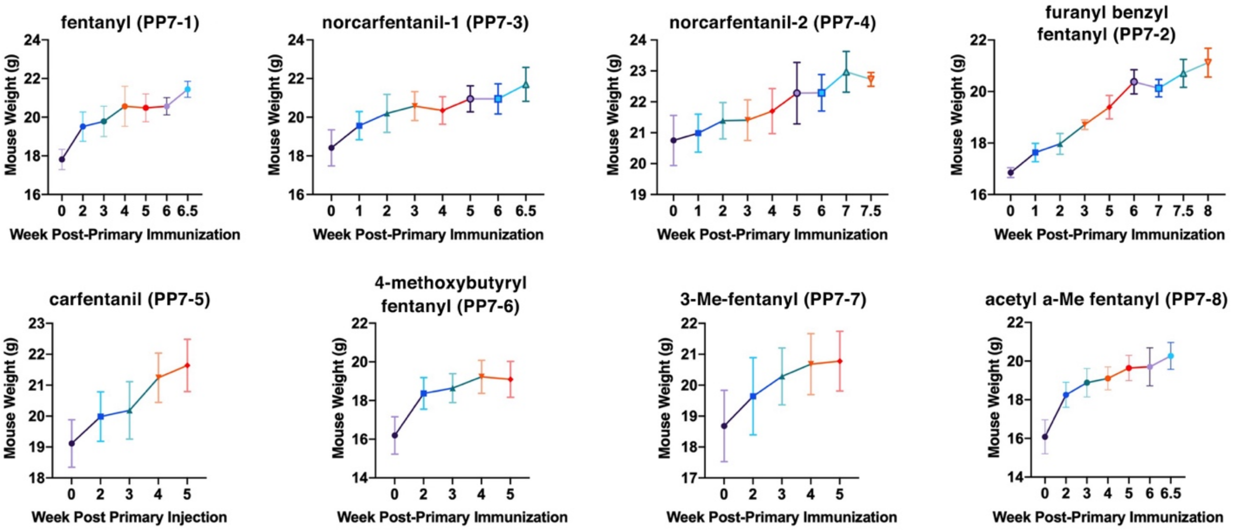

Figure S5. Immunization with PP7-Fentanyl conjugate vaccines is not toxic in mice. Six week old female BALB/c mice were immunized on week 0 with 50 ug of VLP-fentanyl conjugates, followed by boosts at week 2 and 4 prior to sacrifice at week 5 or 6.5 post-primary immunization. Mice were weighed each week as a metric of vaccine safety. Plotted points represent mean of all immunized mice, error bars are standard deviation (n=7 per group, except **PP7-2**, n=3).

IgM response

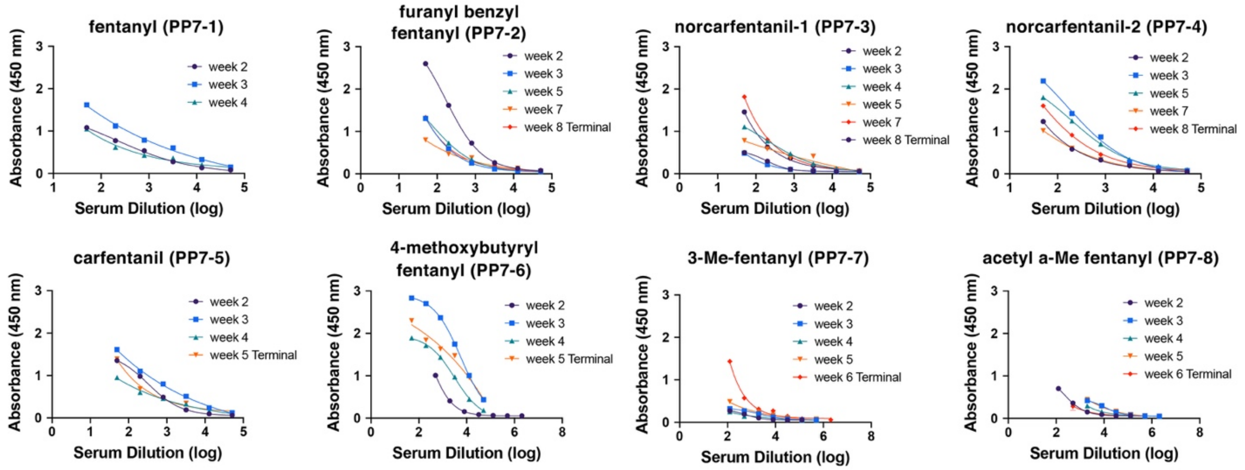

Figure S6. Negligible anti-fentanyl IgM produced in mice immunized with VLP-fentanyl vaccines. Serum from individual immunized mice was pooled at each week of the vaccination series and analyzed for IgM against the corresponding biotinylated fentanyl analog (serum dilutions beginning at 1:50, 6-point titration of 4x serial dilution); n=7 for all except **PP7-2** (n=3).

Non-reactivity with naloxone

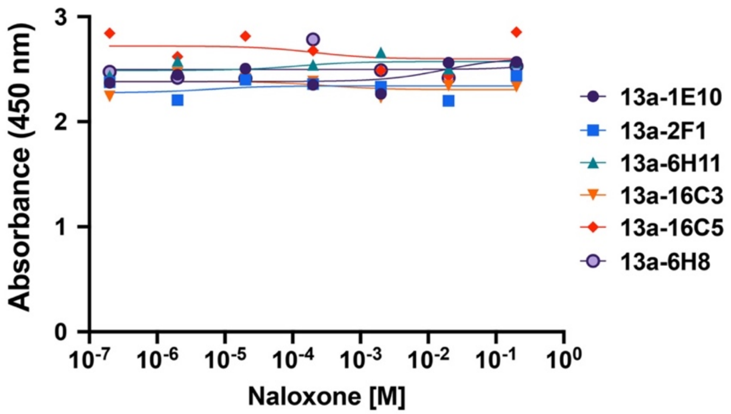

Figure S7. Vaccine-induced anti-fentanyl mAbs do not bind naloxone. Supernatants from antigen-specific hybridomas elicited by vaccination with PP7-2 were screened for naloxone binding by competition ELISA. Streptavidin-coated plates were incubated with biotin-furanyl benzyl fentanyl 10. The indicated supernatants (diluted 1:20 in buffer) were incubated with 10x serially diluted concentrations of naloxone hydrochloride (starting at the saturated concentration of 0.2 M) and then applied to the ELISA plate. After one hour, plates were washed and treated with anti-mouse IgG HRP (1:3000) for one hour. After final washing, TMB substrate was added for 30 sec then quenched with 2M H_2SO_4 and absorbance read at 450 nm by plate reader. Retention of the signal indicates no competition by the added molecule, even up to 0.5M concentration, for antibody binding to the plated antigen.

Antibody binding patterns from immunization with furanyl benzyl fentanyl hapten (2)

The analytes are grouped here into structural classes (Fig. S9) identified in an earlier publication,[7] distinguished by the position and type of variation in the parent fentanyl structure. Excluding these Class 2 and Class 7 analytes, all the other structures were bound by a subset of antibodies, which could be classified into three groups according to their analyte recognition patterns. Group A (7 mAbs) showed cross-reactivity to all of the remaining fentanyl derivatives, with variable affinity for two relatively polar structures (tetrahydrofuranyl in Class 3 and 4-methoxy butyryl fentanyl in Class 4). Group B (5 mAbs) recovered binding ability to the polar analytes but lost affinity for several other analogues (valeryl, phenyl, 4-fluoroisobutyryl, acetyl a-methyl, and norfentanyl, and significant loss of binding ability to b-hydroxythio fentanyl). Group C (9 mAbs) demonstrated variable binding properties, sharing the ability to recognize only the parent fentanyl, the furanyl benzyl hapten used in immunization, and benzyl and *o*-fluoro derivatives.

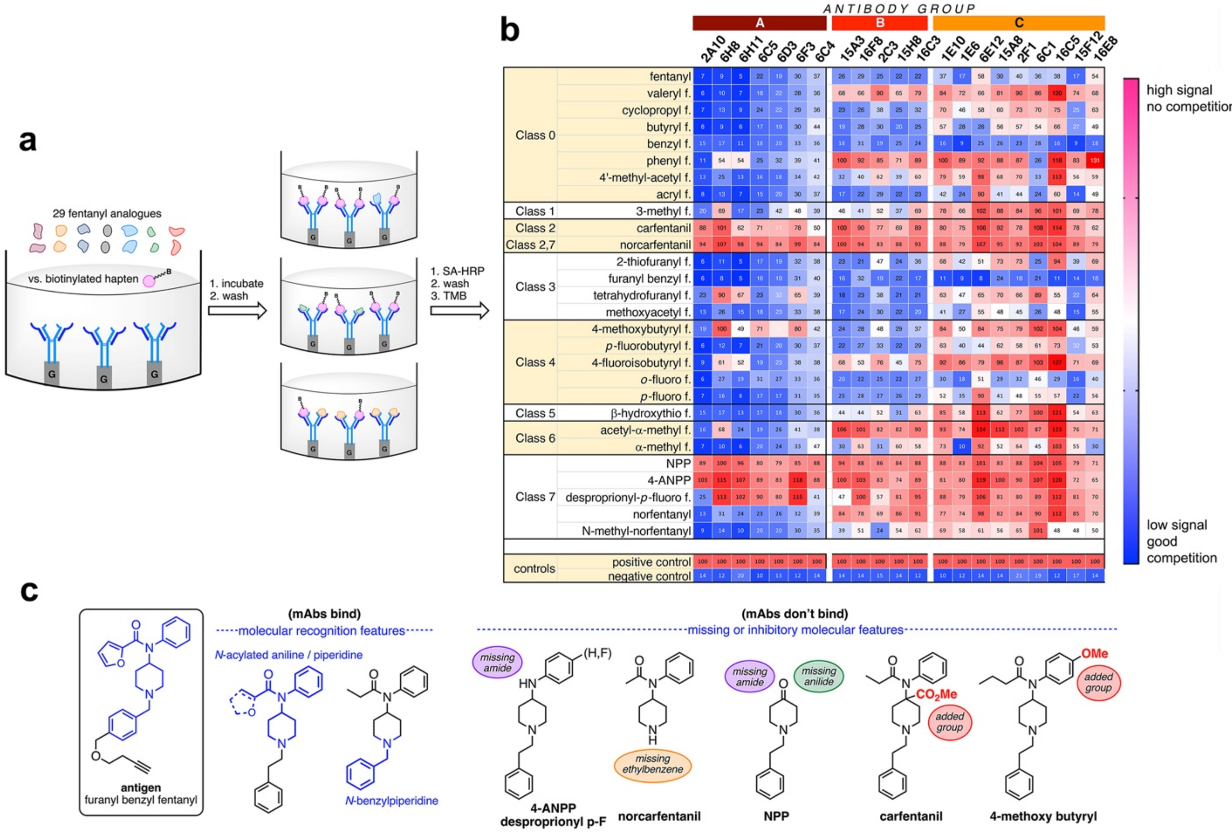

Figure S8. Recognition of fentanyl derivatives by a panel of antibodies raised against furanyl benzyl fentanyl. **a)** ELISA experiment competing reagent **10** against unmodified fentanyl analogues (assayed individually) using a panel of mAbs produced by immunization with PP7-**2**, bearing the furanyl benzyl fentanyl hapten, and independently confirmed to bind well to immobilized **10**. **b)** Competition ELISA results using antibodies (plated from hybridoma supernatants at a 1 µg/mL antibody) and reagent **10** (4 ng/mL) mixed with each of 28 unmodified fentanyl analogues (0.2 µg/mL, approx. 50 times the concentration of **10**), and reported as the percent of biotinylated **10** bound relative to the signal generated in the absence of competitor. The antibodies were organized into four groups based on their binding profiles, as described in the text. Results are normalized averages of three independent runs. SA-HRP = streptavidin - horseradish peroxidase conjugate. **c)** Molecular correlates of the binding results shown in panel **b**. Structures of all of the fentanyl analogues are shown in Fig. S8.

Fentanyl derivative structure classifications

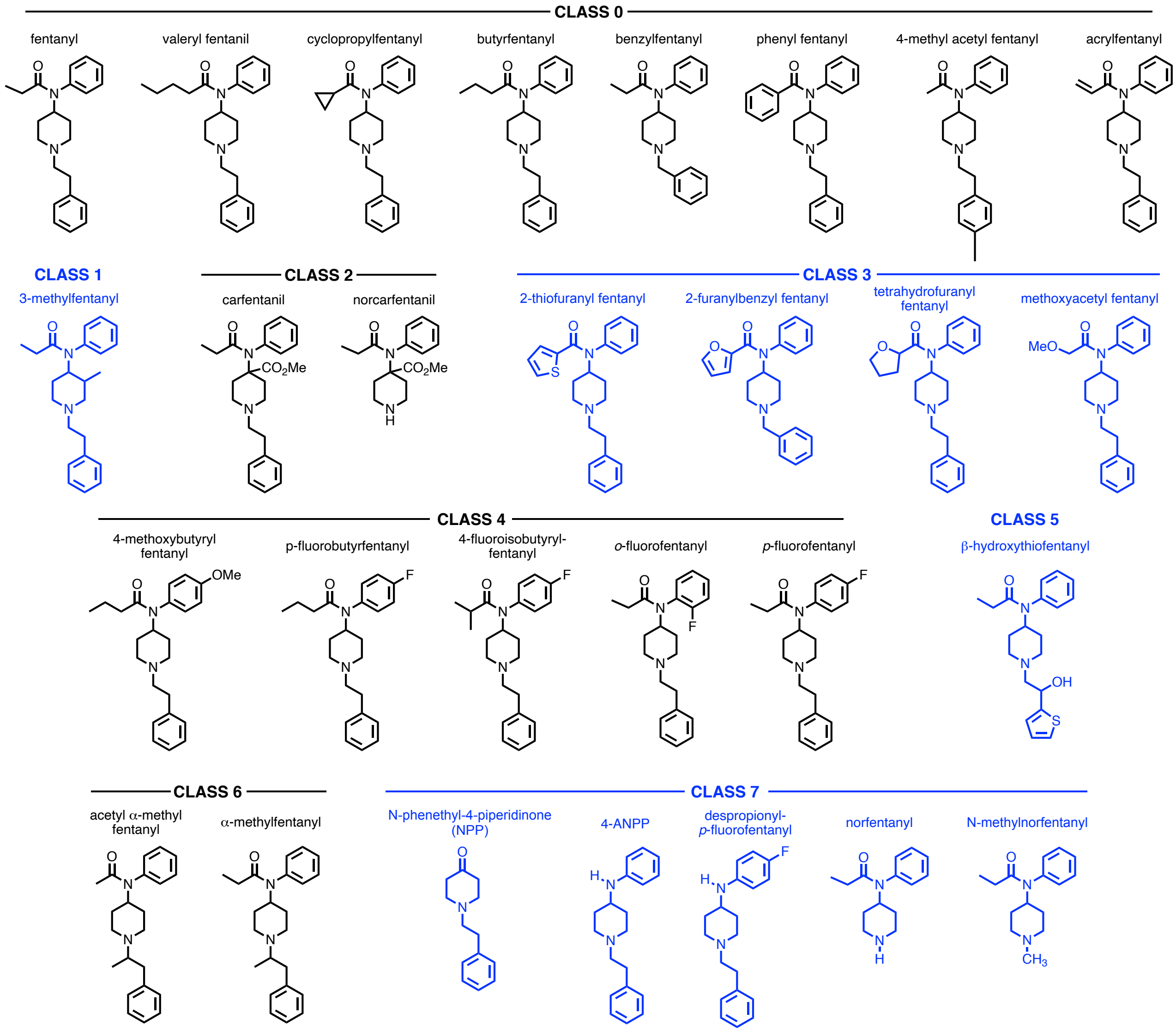

Figure S9. Structures of fentanyl analogues, shown in the order listed in Figure 5. Class designations correspond to structure classifications presented in reference 17.

Hybridoma selection of fentanyl- vs. PP7-specific clones

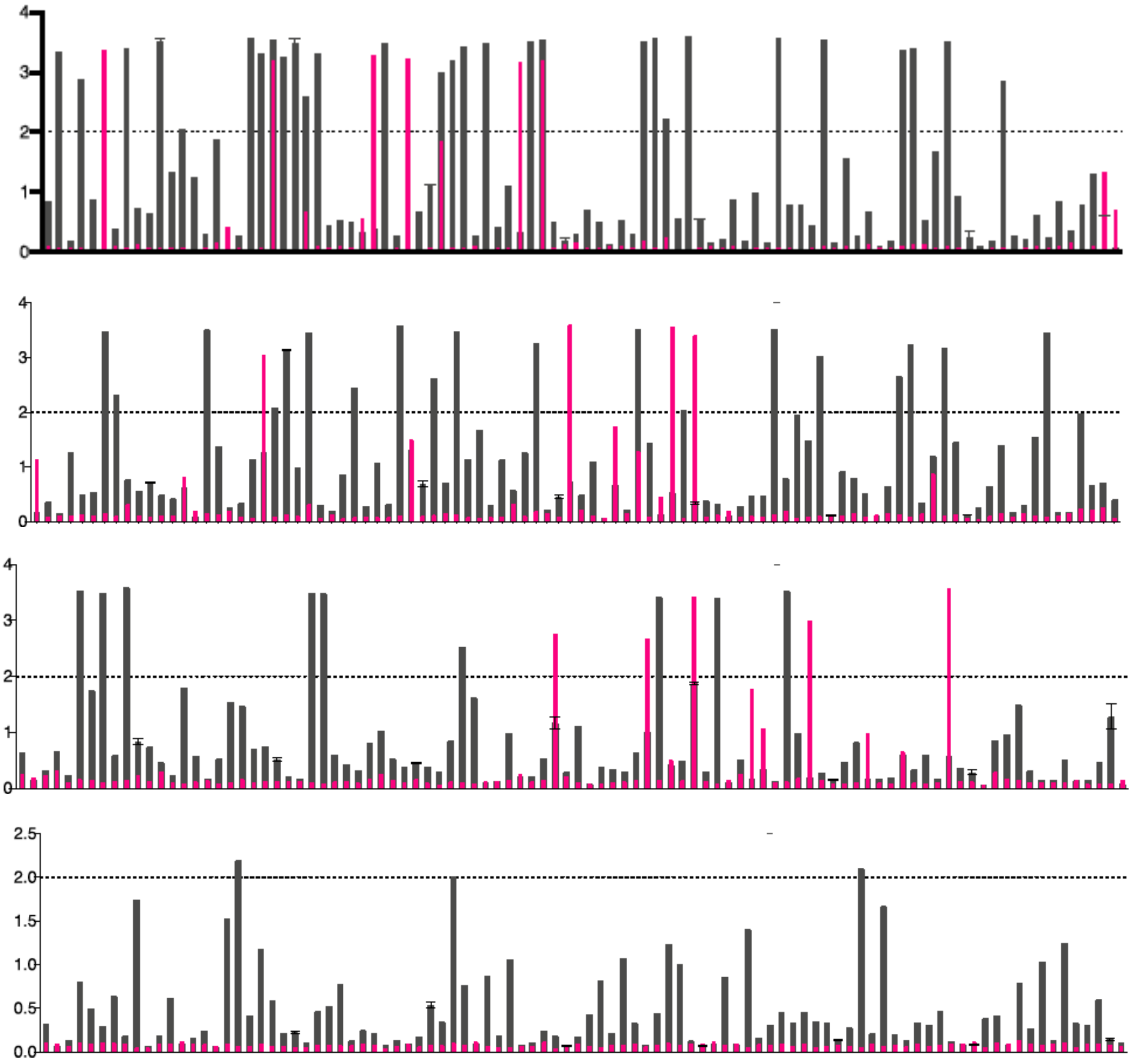

Figure S10. Representative ELISA screening of hybridoma supernatants for binding to plated biotinylated fentanyl (gray) and unfunctionalized PP7 (red).

Screening of hybridoma supernatants

Supernatants vs. purified antibodies

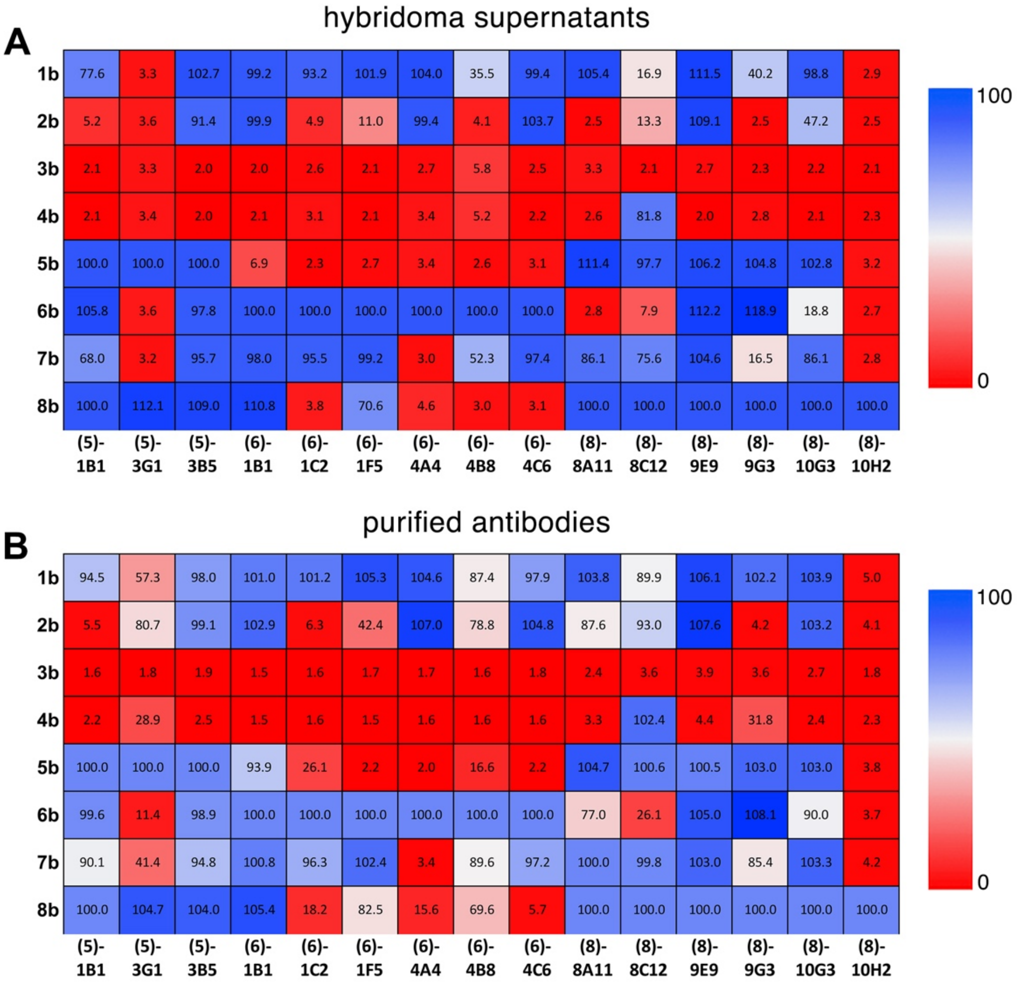

Figure S11. Fentanyl analogue cross reactivity of select clones, showing that supernatants accurately reflect binding patterns of purified antibodies. Relative ELISA results using plated biotinylated antigens with (a) supernatants (1:5 dilution in blocking buffer) from antigen-specific hybridoma clones and (b) purified monoclonal antibodies (5 µg/mL) selected from PP7-8 (αMe), PP7-6 (4MeO), and PP7-5 (carfentanil-1) immunizations. Absorbance was normalized relative to signal against each mAb's cognate antigen signal. Higher values indicate stronger recognition.

Patterns of Fentanyl Analogue Recognition

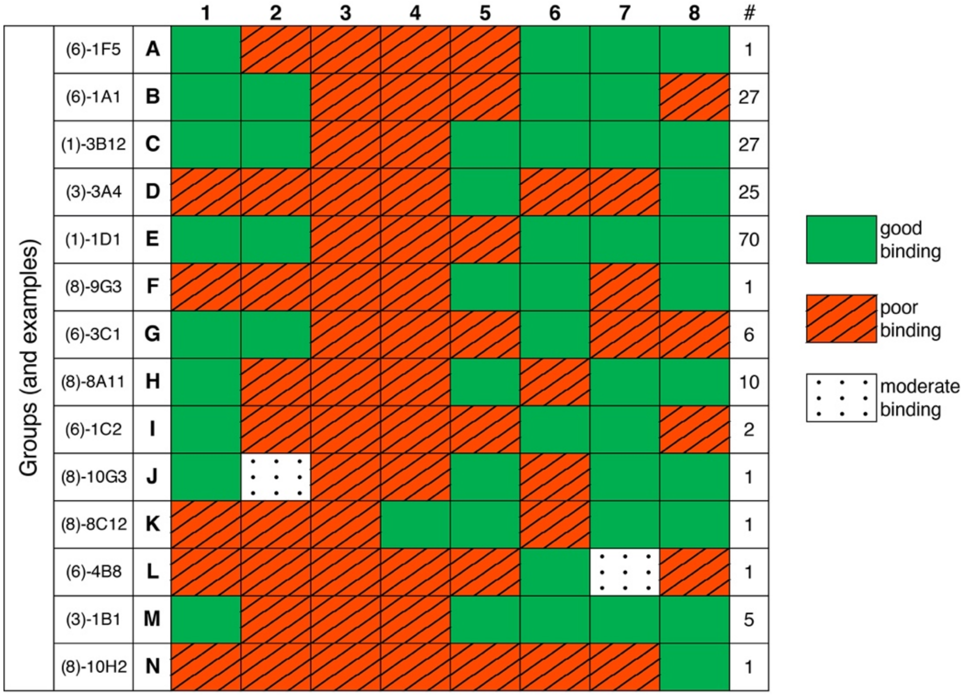

Figure S12. Patterns of fentanyl analogue recognition. Fourteen different patterns (A-N) of binding to immobilized biotinylated derivaitves **1b**-**8b** determined by ELISA (complete results in Table S1). The "#" column reports the number of antibodies found in each group among those shown in Table S1.

Screening of 180 selected clones

Table S1. Screening of hybridoma supernatants for binding to biotinylated fentanyl derivatives. Each biotinylated antigen (0.25 µg/mL) was immobilized to streptavidin-coated ELISA plates by incubation at room temperature for 1 hour, followed by washing once with buffer, blocking with 1% (w/v) BLOK^{TM} casein (G-Biosciences) for 2 hours, and washing twice with buffer. Each supernatant was adjusted to a total protein concentration of 1 mg/mL, and diluted 10x before applying to each well (100 µL). After incubation for 1 hour at room temperature, the wells were washed with buffer, treated with secondary anti-mouse IgG antibody fused to horseradish peroxidase (Southern Biotech) diluted (1:2500) in blocking buffer for 1 hour with shaking, followed by washing. Plates were developed by adding 1-step Ultra TMB (Fisher Scientific) for 90 seconds, followed by quenching with 2 N H_2SO_4. Absorbance (450 nm) was measured by plate reader (Varioskan Flash, Thermo Fisher Scientific). Each 96-well plate contained all eight biotinylated targets (**1b** – **8b**, one per row) treated with supernatants from 12 different hybridomas (columns). These conditions gave a maximum signal (OD_{450}) for all plates of 3.0 ± 0.2. The results for each plate were converted to percentages of the maximum signal to allow for comparison between all of the results. Strong antigen recognition was judged to be represented by signals >75%, medium by signals between 35%-75%, and weak to no recognition by signals <35% of the maximum. Negative (unrelated mouse IgG) controls gave consistent values of < 2%. The "group" designation refers to binding strong/weak patterns shown in Fig. S11, "sim" (similar) means that the pattern (A-M) is assigned by counting a medium binding result as a strong one.

	biotinylated ELISA reagent								
	**1b**	**2b**	**3b**	**4b**	**5b**	**6b**	**7b**	**8b**	**group**
**(1)-1A1**	82.1%	90.1%	2.0%	1.8%	4.8%	81.9%	86.5%	49.9%	E-sim
**(1)-1A11**	78.2%	74.1%	2.6%	1.8%	6.0%	75.6%	75.8%	79.4%	E-sim
**(1)-1A4**	84.8%	80.0%	2.7%	2.0%	69.3%	82.7%	78.8%	91.3%	C-sim
**(1)-1A7**	75.8%	74.4%	1.8%	1.7%	4.1%	81.1%	76.2%	86.6%	E-sim
**(1)-1B10**	79.8%	81.7%	2.1%	1.8%	18.6%	79.6%	82.6%	50.4%	E-sim
**(1)-1B12**	81.4%	81.1%	2.2%	1.9%	11.7%	81.6%	86.7%	63.7%	E-sim
**(1)-1B3**	88.1%	86.9%	2.0%	1.8%	5.0%	81.3%	64.1%	58.2%	E-sim
**(1)-1C12**	79.0%	74.3%	1.9%	1.7%	4.5%	78.9%	74.7%	85.9%	E-sim
**(1)-1C7**	80.5%	76.9%	2.4%	2.6%	15.1%	81.5%	77.0%	91.6%	E
**(1)-1D1**	82.3%	85.9%	2.4%	1.8%	3.8%	84.8%	83.4%	95.0%	E
**(1)-1E5**	86.1%	77.4%	2.0%	1.7%	18.4%	88.1%	84.3%	86.1%	E
**(1)-1F1**	78.0%	76.2%	2.1%	2.1%	25.0%	88.2%	74.6%	97.5%	E
**(1)-1F12**	80.0%	76.2%	2.3%	1.8%	23.4%	81.7%	76.2%	85.1%	E
**(1)-1F5**	76.3%	77.6%	1.8%	1.8%	5.7%	78.7%	79.9%	81.5%	E
**(1)-1G5**	79.7%	76.2%	1.9%	1.7%	32.2%	79.8%	78.7%	86.6%	E
**(1)-1G7**	84.4%	81.4%	2.4%	2.1%	32.1%	86.9%	84.2%	87.6%	E
**(1)-1H5**	78.2%	84.8%	1.9%	1.6%	4.8%	81.3%	83.1%	57.4%	E-sim
**(1)-2A6**	84.2%	83.4%	2.3%	2.0%	7.7%	95.9%	82.9%	96.7%	E
**(1)-2D12**	84.4%	81.7%	2.9%	2.3%	80.7%	82.1%	83.8%	90.4%	C
**(1)-2F2**	87.7%	95.9%	2.5%	2.1%	16.5%	89.9%	93.2%	74.7%	E
**(1)-3B10**	88.0%	82.0%	1.8%	1.6%	5.6%	70.5%	61.1%	66.4%	E-sim
**(1)-3B12**	83.7%	85.1%	2.1%	1.8%	82.4%	86.6%	83.3%	89.9%	3C
**(1)-3F3**	88.0%	86.7%	3.0%	2.5%	35.1%	85.0%	86.5%	90.1%	C-sim
**(1)-3H11**	84.0%	85.9%	2.3%	2.0%	23.9%	85.1%	80.8%	69.6%	E-sim
**(1)-4A1**	80.0%	77.0%	2.3%	2.0%	3.5%	79.8%	78.3%	84.6%	5E

Table S1, continued.

	biotinylated ELISA reagent								
	**1b**	**2b**	**3b**	**4b**	**5b**	**6b**	**7b**	**8b**	**group**
**(1)-4B2**	81.8%	83.1%	2.0%	1.7%	50.2%	87.5%	81.1%	89.0%	C-sim
**(1)-4D8**	84.2%	82.4%	2.4%	2.0%	98.9%	83.6%	82.1%	82.1%	C
**(1)-4F12**	79.7%	85.0%	2.1%	1.8%	17.9%	84.4%	81.9%	87.8%	E
**(1)-4G11**	77.1%	79.4%	1.9%	1.6%	3.3%	78.4%	78.0%	85.7%	E
**(1)-4G3**	81.2%	86.0%	2.2%	3.4%	2.5%	84.4%	78.0%	91.1%	E
**(1)-4G5**	83.9%	87.8%	2.9%	2.0%	2.8%	85.4%	88.4%	15.5%	B
**(1)-4H1**	80.0%	78.3%	2.0%	1.5%	8.5%	78.5%	79.8%	90.8%	E
**(1)-4H11**	84.5%	87.8%	2.4%	1.9%	3.6%	89.4%	88.9%	56.9%	E-sim
**(1)-5A1**	84.9%	89.2%	2.3%	2.0%	2.9%	85.7%	88.1%	4.8%	B
**(1)-5A11**	85.1%	91.6%	2.4%	2.0%	16.5%	88.0%	93.0%	58.6%	E-sim
**(1)-5A6**	84.2%	81.5%	2.2%	1.8%	19.9%	82.2%	83.9%	75.0%	E
**(1)-5D1**	83.5%	81.4%	2.0%	1.8%	8.5%	84.0%	82.0%	84.2%	E
**(1)-5D2**	82.9%	80.5%	2.1%	2.0%	41.8%	88.1%	81.0%	91.9%	C-sim
**(1)-5F1**	82.6%	85.1%	2.4%	2.0%	12.4%	84.9%	83.7%	90.4%	E
**(1)-5F12**	79.7%	78.8%	2.0%	1.8%	5.4%	78.0%	78.3%	86.4%	E
**(1)-5F2**	82.2%	84.1%	2.3%	2.0%	13.7%	83.1%	83.0%	90.0%	E
**(1)-5F4**	86.0%	83.8%	3.0%	2.5%	49.9%	87.7%	84.0%	93.3%	C-sim
**(1)-5F5**	87.5%	84.5%	2.1%	1.8%	22.4%	88.8%	88.4%	63.7%	E-sim
**(1)-5F6**	87.0%	83.3%	2.5%	2.2%	7.7%	83.7%	84.5%	86.0%	E
**(1)-5F7**	86.1%	85.0%	2.8%	2.0%	34.3%	80.0%	86.2%	86.1%	E
**(1)-5F8**	86.3%	82.9%	3.0%	1.8%	5.6%	86.1%	88.9%	47.2%	E-sim
**(1)-5H10**	85.8%	87.0%	2.1%	2.1%	8.4%	88.8%	83.8%	94.4%	E
**(1)-5H2**	81.8%	77.9%	2.2%	1.9%	3.6%	78.5%	84.3%	86.3%	E
**(1)-5H3**	83.9%	88.5%	2.1%	3.0%	16.7%	87.7%	84.1%	94.0%	E
**(1)-6A1**	80.4%	78.7%	2.6%	1.9%	12.6%	76.6%	79.9%	87.0%	E
**(1)-6C2**	87.3%	91.5%	2.6%	2.2%	4.5%	86.3%	89.4%	43.0%	E-sim
**(1)-6C7**	87.3%	88.1%	2.6%	2.2%	29.3%	90.4%	88.2%	90.9%	E
**(1)-6D3**	89.5%	92.2%	2.2%	2.3%	3.9%	91.7%	86.8%	37.1%	E-sim
**(1)-6E1**	80.0%	87.6%	2.1%	1.9%	16.8%	52.2%	84.1%	74.3%	E-sim
**(1)-6E2**	95.1%	90.1%	2.1%	1.9%	2.2%	88.7%	88.1%	54.9%	E-sim
**(1)-6F1**	86.6%	91.8%	2.3%	2.0%	2.7%	87.5%	85.8%	14.2%	B
**(1)-6G5**	82.0%	85.7%	2.5%	2.2%	5.4%	84.3%	83.4%	91.7%	E
**(1)-6H6**	84.6%	90.0%	2.9%	2.6%	26.1%	87.2%	90.7%	57.4%	E-sim
**(1)-6H7**	82.4%	82.5%	2.9%	2.7%	91.6%	85.7%	84.7%	89.3%	C
**(3)-1B1**	68.6%	4.6%	1.9%	1.9%	88.4%	93.5%	60.1%	88.4%	M-sim

Table S1, continued.

	biotinylated ELISA reagent								
	**1b**	**2b**	**3b**	**4b**	**5b**	**6b**	**7b**	**8b**	**group**
**(3)-3A4**	8.5%	3.5%	1.8%	1.9%	87.3%	6.1%	29.0%	99.9%	D
**(3)-3A5**	2.3%	2.6%	2.0%	1.9%	85.5%	2.5%	2.7%	87.0%	D
**(3)-3B1**	2.6%	4.3%	1.7%	1.8%	86.4%	7.2%	3.5%	94.0%	D
**(3)-3B5**	94.2%	83.9%	1.8%	1.8%	91.8%	89.8%	87.8%	100.0%	C
**(3)-3E4**	8.1%	3.2%	1.9%	1.8%	95.9%	4.0%	30.5%	91.6%	D
**(3)-3F1**	3.5%	12.1%	2.7%	2.8%	87.5%	6.1%	2.8%	80.3%	D
**(3)-3G1**	2.9%	3.1%	2.8%	3.0%	87.4%	3.1%	2.8%	98.0%	D
**(3)-3G3**	2.8%	3.2%	2.4%	2.4%	82.9%	2.7%	2.7%	71.0%	D-sim
**(3)-3H1**	2.9%	3.3%	2.0%	1.7%	85.7%	3.3%	3.0%	88.1%	D
**(6)-1A1**	77.8%	81.3%	3.7%	2.1%	2.2%	77.3%	83.3%	3.3%	B
**(6)-1A2**	87.4%	8.8%	1.9%	1.8%	2.7%	86.8%	48.4%	3.7%	I-sim
**(6)-1A4**	83.6%	84.1%	3.1%	2.2%	2.4%	78.6%	87.3%	4.2%	B
**(6)-1A5**	79.1%	83.0%	3.7%	2.0%	2.5%	83.4%	82.3%	4.3%	B
**(6)-1B1**	89.6%	90.2%	1.8%	1.9%	6.3%	90.3%	88.5%	100.0%	E
**(6)-1B3**	83.8%	82.9%	1.9%	1.8%	2.8%	80.8%	83.5%	4.3%	E
**(6)-1B5**	79.9%	78.8%	3.0%	1.9%	2.9%	78.6%	86.7%	6.6%	B
**(6)-1C2**	77.6%	4.0%	2.2%	2.6%	1.9%	83.3%	79.6%	3.2%	I
**(6)-1D2**	87.4%	44.7%	1.9%	1.8%	1.9%	80.5%	16.2%	3.8%	G-sim
**(6)-1D6**	77.1%	80.9%	3.1%	1.8%	3.1%	87.2%	82.8%	92.9%	E
**(6)-1D9**	81.4%	66.0%	2.9%	2.1%	2.3%	82.2%	88.9%	35.3%	E-sim
**(6)-1E2**	99.8%	42.1%	1.8%	2.1%	2.1%	85.2%	13.8%	3.1%	G-sim
**(6)-1E4**	84.5%	86.0%	4.2%	2.0%	14.4%	81.0%	80.6%	20.0%	2
**(6)-1F2**	92.4%	48.2%	2.0%	1.9%	2.1%	92.7%	20.7%	3.3%	G-sim
**(6)-1F5**	90.3%	9.7%	1.9%	1.9%	2.4%	88.6%	87.9%	62.6%	A
**(6)-1G4**	85.6%	86.1%	2.8%	2.0%	2.4%	88.9%	86.1%	96.0%	E
**(6)-1H1**	81.5%	87.0%	1.9%	1.8%	2.0%	85.8%	75.9%	5.1%	B
**(6)-1H8**	85.4%	88.7%	2.2%	2.0%	2.8%	86.0%	87.6%	4.2%	B
**(6)-2B2**	82.4%	89.1%	5.7%	2.5%	5.1%	82.1%	81.6%	5.3%	B
**(6)-2C1**	84.2%	92.9%	2.1%	1.8%	17.6%	83.9%	88.8%	97.2%	E
**(6)-2D2**	80.6%	89.1%	6.5%	2.0%	2.2%	81.2%	83.6%	3.8%	B
**(6)-2F5**	86.2%	89.7%	2.2%	2.0%	4.4%	88.9%	88.1%	100.0%	E
**(6)-3C1**	83.4%	89.1%	2.2%	1.8%	3.3%	85.3%	25.0%	9.9%	G
**(6)-3C2**	81.5%	84.3%	2.1%	2.0%	2.4%	87.8%	83.8%	8.7%	B
**(6)-3D1**	63.1%	54.7%	4.3%	1.8%	2.2%	84.0%	72.1%	2.6%	E-sim
**(6)-3D5**	82.0%	94.3%	4.0%	2.1%	2.7%	84.4%	83.2%	2.5%	B
**(6)-3E2**	80.9%	82.7%	1.8%	1.9%	1.9%	82.5%	81.0%	2.3%	B

Table S1, continued.

	biotinylated ELISA reagent								
	**1b**	**2b**	**3b**	**4b**	**5b**	**6b**	**7b**	**8b**	**group**
**(6)-3E4**	80.9%	84.4%	1.9%	1.7%	2.0%	82.2%	81.7%	2.5%	B
**(6)-3F5**	81.0%	82.2%	2.6%	2.1%	5.5%	83.0%	78.8%	10.4%	B
**(6)-4A2**	84.4%	82.2%	4.5%	4.6%	95.6%	83.6%	80.6%	97.2%	C
**(6)-4A4**	86.1%	82.3%	2.3%	2.8%	2.8%	82.8%	2.5%	3.8%	G
**(6)-4B1**	91.3%	94.9%	9.4%	10.5%	9.2%	86.6%	87.2%	3.3%	B
**(6)-4B3**	82.3%	81.8%	2.1%	3.7%	2.8%	81.2%	2.0%	3.8%	G
**(6)-4B6**	84.6%	63.2%	5.8%	6.3%	7.7%	82.5%	84.5%	2.5%	B-sim
**(6)-4B8**	30.7%	3.5%	5.0%	4.5%	2.3%	86.6%	45.3%	2.6%	L
**(6)-4C2**	92.4%	80.6%	6.0%	13.8%	36.6%	83.0%	54.7%	75.4%	C-sim
**(6)-4C6**	86.0%	89.7%	2.2%	1.9%	2.7%	86.5%	84.2%	2.7%	B
**(6)-4D4**	82.9%	82.6%	2.4%	2.0%	4.5%	83.4%	81.8%	3.1%	B
**(6)-4E1**	87.5%	63.0%	2.2%	2.1%	9.4%	85.5%	89.4%	13.2%	B-sim
**(6)-4E4**	92.0%	84.8%	2.3%	1.9%	13.9%	81.4%	94.7%	3.5%	B
**(6)-4E5**	83.0%	73.6%	1.8%	2.0%	10.8%	83.7%	86.4%	2.7%	B-sim
**(6)-4E6**	84.5%	69.3%	1.8%	1.8%	7.1%	85.4%	88.3%	2.7%	B-sim
**(6)-4F3**	85.5%	88.6%	2.4%	1.8%	3.7%	81.9%	84.2%	81.8%	E
**(6)-4G2**	90.1%	84.6%	2.2%	1.7%	6.0%	82.8%	84.3%	85.7%	E
**(6)-4H5**	86.0%	52.0%	1.8%	1.7%	2.5%	83.4%	89.8%	8.7%	B-sim
**(7)-1A2**	83.1%	80.1%	5.9%	2.7%	74.8%	80.1%	79.9%	83.1%	C
**(7)-1G1**	78.5%	78.2%	5.3%	2.2%	68.8%	78.7%	76.0%	83.0%	C-sim
**(7)-1H2**	77.8%	77.7%	2.7%	2.5%	4.8%	86.2%	80.3%	93.1%	E
**(7)-2A1**	71.9%	68.3%	2.3%	1.9%	2.3%	71.1%	76.7%	89.3%	E-sim
**(7)-2A2**	77.0%	76.9%	7.4%	3.7%	77.4%	71.8%	76.3%	70.8%	C-sim
**(7)-2A6**	75.7%	76.6%	2.4%	1.7%	2.6%	79.1%	77.5%	84.6%	E
**(7)-2A8**	58.8%	48.2%	1.6%	1.9%	2.9%	49.5%	52.5%	41.4%	E-sim
**(7)-2B4**	80.2%	79.1%	2.6%	1.8%	3.6%	76.1%	80.4%	90.5%	E
**(7)-2B5**	65.2%	61.2%	1.9%	2.0%	72.8%	74.7%	65.8%	62.9%	C-sim
**(7)-2C1**	78.9%	74.8%	2.4%	2.0%	34.6%	85.0%	75.4%	87.4%	E
**(7)-2C2**	75.1%	73.0%	8.4%	2.5%	42.6%	73.8%	74.6%	78.0%	C-sim
**(7)-2C3**	79.0%	72.4%	2.4%	1.8%	2.5%	84.1%	77.3%	82.0%	E-sim
**(7)-2C7**	63.0%	62.3%	1.9%	1.6%	2.3%	71.5%	67.5%	55.0%	E-sim
**(7)-2D2**	74.8%	73.2%	2.2%	1.7%	2.3%	86.5%	73.9%	84.7%	E-sim
**(7)-2D3**	69.4%	85.0%	9.7%	2.2%	100.0%	81.8%	80.7%	86.8%	C-sim
**(7)-2F2**	79.5%	84.3%	3.6%	1.9%	81.6%	86.1%	84.2%	93.6%	C
**(7)-2F3**	80.7%	83.1%	4.3%	2.1%	30.6%	84.0%	80.7%	89.8%	E
**(7)-2F5**	84.6%	83.1%	2.6%	1.9%	79.9%	89.0%	83.6%	92.3%	E

Table S1, continued.

	biotinylated ELISA reagent								
	**1b**	**2b**	**3b**	**4b**	**5b**	**6b**	**7b**	**8b**	**group**
**(7)-2G1**	87.7%	88.0%	1.8%	1.8%	87.9%	98.3%	82.9%	90.7%	C
**(7)-2G2**	81.0%	83.8%	2.8%	2.2%	20.0%	83.5%	85.2%	91.2%	E
**(7)-2G3**	4.3%	17.5%	1.7%	1.7%	2.3%	5.3%	5.5%	12.4%	--
**(7)-2G9**	61.1%	68.8%	2.1%	2.1%	2.7%	77.8%	67.9%	82.2%	E-sim
**(7)-2H1**	54.4%	68.1%	3.1%	2.2%	2.4%	66.2%	64.6%	80.7%	E-sim
**(7)-2H2**	78.8%	79.0%	5.5%	2.5%	53.9%	84.8%	84.1%	95.9%	C-sim
**(7)-2H3**	82.7%	86.0%	2.2%	3.4%	3.3%	82.2%	62.6%	88.6%	E-sim
**(7)-2H5**	83.5%	82.7%	2.8%	1.8%	37.4%	87.4%	79.9%	91.7%	C-sim
**(8)-10D3**	3.0%	3.2%	2.1%	1.8%	6.4%	3.2%	2.3%	14.3%	--
**(8)-10G3**	88.4%	42.2%	1.9%	1.9%	92.0%	16.8%	77.0%	89.5%	J
**(8)-10H2**	2.5%	2.1%	1.8%	2.0%	2.8%	2.3%	2.4%	86.0%	N
**(8)-7A7**	6.0%	9.1%	1.7%	1.8%	86.3%	6.4%	10.6%	83.0%	D
**(8)-8A11**	85.6%	2.0%	2.7%	2.1%	90.5%	2.3%	69.9%	81.2%	H
**(8)-8A2**	62.5%	57.0%	1.7%	1.7%	84.4%	57.5%	61.3%	81.2%	C-sim
**(8)-8C12**	14.0%	11.0%	1.8%	67.6%	80.8%	6.5%	62.5%	82.7%	K
**(8)-8H5**	66.7%	25.0%	1.7%	1.8%	91.1%	30.6%	80.0%	83.3%	H
**(8)-9A1**	7.0%	5.0%	1.8%	4.2%	80.5%	24.5%	5.9%	81.8%	D
**(8)-9A2**	75.0%	7.0%	1.9%	1.9%	87.0%	35.3%	72.7%	84.2%	M-sim
**(8)-9A3**	12.3%	4.6%	1.8%	1.8%	53.2%	32.1%	13.1%	83.5%	D-sim
**(8)-9A5**	70.0%	11.9%	2.1%	5.5%	81.4%	12.4%	72.1%	82.3%	H
**(8)-9A7**	4.8%	20.2%	1.8%	2.7%	29.5%	10.5%	4.5%	82.5%	N
**(8)-9B3**	86.2%	84.5%	1.9%	1.9%	94.1%	87.7%	85.9%	83.3%	C
**(8)-9B4**	27.5%	24.9%	2.1%	2.0%	82.1%	32.8%	28.7%	79.3%	D
**(8)-9B5**	66.5%	2.3%	1.8%	1.7%	83.7%	17.0%	67.7%	84.2%	H
**(8)-9C2**	92.6%	13.2%	1.9%	1.8%	87.7%	56.7%	89.1%	56.2%	M-sim
**(8)-9C3**	10.1%	4.1%	1.9%	1.9%	86.8%	3.1%	5.5%	83.2%	D
**(8)-9C5**	13.9%	12.8%	1.9%	1.9%	87.5%	5.2%	13.9%	81.6%	D
**(8)-9C6**	3.7%	2.4%	1.9%	2.2%	82.6%	10.7%	3.5%	82.4%	D
**(8)-9D2**	3.0%	2.1%	1.9%	1.9%	84.3%	4.1%	2.6%	55.8%	D-sim
**(8)-9D6**	81.4%	2.2%	1.9%	3.2%	93.3%	91.7%	65.9%	61.1%	M-sim
**(8)-9E4**	40.8%	32.0%	1.9%	1.9%	89.8%	16.8%	41.4%	59.2%	H
**(8)-9E5**	83.0%	2.5%	1.9%	7.8%	85.6%	9.6%	65.7%	83.4%	H
**(8)-9E8**	57.3%	3.9%	1.8%	1.8%	89.8%	2.8%	58.9%	84.9%	H
**(8)-9E9**	93.1%	91.1%	2.3%	1.7%	88.7%	93.7%	87.4%	83.5%	C

Table S1, continued.

	biotinylated ELISA reagent								
	**1b**	**2b**	**3b**	**4b**	**5b**	**6b**	**7b**	**8b**	**group**
**(8)-9F1**	2.7%	2.5%	2.2%	1.9%	91.1%	2.3%	2.9%	85.8%	C-sim
**(8)-9F2**	51.5%	38.4%	2.5%	2.0%	89.4%	22.3%	48.8%	84.3%	D-sim
**(8)-9F3**	13.9%	2.5%	2.0%	1.9%	87.2%	3.6%	13.8%	61.2%	D
**(8)-9F4**	14.5%	5.4%	1.9%	1.8%	90.1%	5.1%	12.0%	87.6%	D
**(8)-9F5**	8.8%	6.5%	2.0%	2.1%	86.0%	10.4%	8.3%	83.4%	D
**(8)-9F6**	12.3%	4.4%	1.9%	2.2%	76.1%	2.5%	10.2%	75.5%	D
**(8)-9F7**	19.9%	3.1%	2.0%	1.9%	83.1%	4.1%	6.1%	79.4%	D
**(8)-9G2**	2.7%	4.2%	1.8%	2.3%	95.7%	2.5%	6.6%	87.2%	D
**(8)-9G3**	33.8%	2.1%	1.9%	2.3%	88.1%	100.0%	13.9%	84.1%	F
**(8)-9G4**	78.8%	19.2%	2.1%	3.5%	85.8%	39.1%	35.9%	83.9%	D
**(8)-9G7**	3.1%	2.2%	2.0%	1.8%	91.8%	32.0%	2.9%	84.7%	H
**(8)-9H4**	37.6%	34.3%	1.9%	2.1%	92.8%	33.7%	39.3%	88.5%	C-sim
**blank**	3.1%	3.6%	2.2%	1.9%	2.1%	3.0%	2.9%	3.0%	
**blank**	2.3%	2.2%	1.9%	2.1%	2.8%	2.2%	2.4%	3.3%	

Sequences of selected clones

Table S2. Complementarity determining region (CDR) and framework region (FR) sequences of selected mAbs, color coded to identify high degrees of similarity separately in light-chain and heavy-chain sequences.

light chain

clone	FR1	CDR1	FR2	CDR2	FR3	CDR3	FR4
**(5)-1B1**	QAVVTQESALTTSPGETVTL TCRSS	TGALTTS NY	ANWVQEKPDHLF TGLIG	GAN	NRAPGVPARFSGSLIGDKAALTI TGAQTEDEAIYF	CALWYSN HLVF	GGGTKLT VL
**(5)-3G1**	DIVMTQSQKFMSTSVGDR VSVTCKAS	QNVGAN	VAWSQQKPGQS PKLLIY	SAS	YRYSGVPDRFTGSGSGTDFTLTIS NVQSEDLAEYF	CQQYNSYP YTF	GGGTKLE IK
**(6)-1F5**	DIVMTQSQKFMSTSVGDR VSVTCKAS	QNVGTN	VVWYQQKPGQS PKALIY	SAS	YRYSGVPDRFTGSGSGTDFTLTIS NVQSEDLAEYF	CQQYNSYP YTF	GGGTKLE IK
**(6)-4C6**	DIVMTQAAFSNPVTLGTSA SISCRSS	KSLLHSN GITY	LYWYLQKPGQSP QLLIY	QMS	NLASGVPDRFSSSGSGTDFTLRIS RVEAEDVGAYY	CAQNLELP WTF	GGGTKLE IK
**(6)-1B1**	DIVMTQSQKFMSTSVGDR VSVTCKAS	QNVGTN	VVWYQQKPGQS PKALIY	SAS	YRYSGVPDRFTGSGSGTDFTLTIS NVQSEDLAEYI	CQQYNSYP LTF	GAGTKLE LK
**(6)-4A4**	DILMTQSPSSMSVSLGDTV SITCHAS	QGISSS	IGWLQQKPGKSF KGLIS	HGT	KLEDGVPSRFSGSGSGADFSLTIS SLESEDLADYY	CVQYAQFP YTF	GGGTKLE MK
**(6)-1C2**	NIVMTQSQKFMSTSVGDR VSVTCKAS	QNVGTN	VAWYQQKPGQS PKALIY	SAS	YQYSGVPDRFTGSGSGTDFTLTI SNVQSEDLAEYF	CQQYNSYP LTF	GAGTKLE LK
**(8)-9E9**	DIVMTQSQKFMSTSVGDR VSVTCKAS	QNVGTN	VGWCQQKPGQS PKALIY	STS	YRYSGVPDRFTGSGSGTDFILTIS NVQSEDLAEYF	CLQYNSYP FTF	GSGTKLE IK
**(8)-9G3**	QAVVTQESALTTSPGETVTL TCRSS	TGAVTSS NY	ANWVQEKPDHLF TGLIG	GTS	NRAPGVPARFSGSLIGDKAALTI TGAQTEDEAIYF	CALWYSN HLVF	GGGTKLT VL
**(8)-8A11**	DIVMTQSQKFMSTSVGDR VSVTCKAS	QNVGTN	VAWYQQKPGQS PKSLIY	LTS	YRYSGVPDRFTGSGSGTDFTLTIR NVQSEDLAEYF	CQQYNSYP FTF	GSGTKLE IK
**(8)-10G3**	DIVMTQSQKFMSTSVGDR VSVTCKAS	QNVGTN	VAWCQQKPGQS PKSLIY	LAS	YRYSGVPDRFTGSGFGTDFTLTIS NVQSEDLAEYF	CLQYDSYP WTF	GGGTKLE IK
**(8)-8C12**	DIVMTQAAFSNPVTLGTSA SMSCRSS	KSLLHSD GITY	LHWYLQKPGQSP QLLIY	QMS	NLASGVPDRFSSSGSGTDFTLRIS RVEAEDVGVYY	CVQNLELP WTF	GGGTKLE IK

heavy chain

clone	FR1	CDR1	FR2	CDR2	FR3	CDR3	FR4
**(5)-1B1**	QVQLQQSGAELMKPG ASVKISCKAS	GYTFSS YW	IEWVKQRPGHGL EWIGE	ILPGSGS T	NYAERFKDKATFTADSSSNTAYM QLTSLTSEDSAVYY	CARFDGNYDSY AMDYW	GQGTSV TVSS
**(5)-3G1**	EVQLEESGGGLVHPGG SMKLSCVAS	GFTFS NSW	MNWVRQSPEKG LEWVAE	IRLKSNN YAT	QFAESVKGRFSISRDDSKSCVYLQ MNNLRAEDTAIYY	CILESAYYTEA YW	GQGTLV TVSA
**(6)-1F5**	EVQLQQSGAELVKPGA SVKLSCTAS	GFNIK DTY	MHWVKQRPEQ GLEWIGR	IDPANG NT	KYDPKFQGKATITADTSSNTAYLQL SSLTSEDTAVYY	CASDGYHLDY W	GQGTTL TVSS
**(6)-4C6**	EVQLQESGPGLVKPSQ TLSLTCSVT	GDSITS GY	WNWIRKFPGNK LEYMGY	INYSGST	YYNPSLKSRISITRDTSKNLYYLQLN SVTADDTATYY	CARYPCDGHN CYIDVW	GAGTTV TVSS
**(6)-1B1**	EVQLQQSGPELVKPGA SVKISCKTS	GYTFT EYT	MHWVKQSHGKS LEWIGS	INPINGG T	SYNQKFKGKATLTVDKSSSTAYME LRSLTSEDSAVYY	CAESGNYDFW	GQGTTL TVSS
**(6)-4A4**	QVQLKESGPGLVPPSQ SLSITCTVS	GFSLTS YG	VHWVRQPPGKG LEWLGV	IWAGGR T	FYNSALMSRLSISKDNSKRQVFLE MNSLQTDDTAMYY	CVRDTDEAYW	GQGTLV TVSA
**(6)-1C2**	DVQLQESGPDLVKPSQ SLSLTCTVT	GYSFTS GYS	WHWIRQFPGNK LEWMGY	ISYDGSN	NYNPSLKGRISITRDTSKNQFFLQL NSVTTEDTATYY	DGATPLTGDY W	GQGTTL TVSS
**(8)-9E9**	EVKVEESGGGLVQPGG SMRLSCVAS	GFTFS NYW	MNWVRQSPEKG LEWVAE	IRLSSNT YAI	HYAESVKGRFTISRDDSKSSVYLQ MNNLRAEDTGIYY	CTREVYRYDEG FAYW	GQGTLV TVSA
**(8)-9G3**	QAQLQQPGTELVRPG ASMKLSCKAS	GYLFTT NW	MSWVKQRPGQ GLEWIGM	IHPSDSE T	RLNQKFKDKATLTVDRSSSTAYMQ LSSPTSEDSAVYY	CARSDDYDVSY W	GHGTLV TVSA
**(8)-8A11**	QVQVQQSGAELVKPG ASVKLSCKAS	GFTFIN SY	MYWVKQRPGQ GLEWIAE	IDLSNG DT	NFNEKFKSKATLTVDKSSSTAYMQ LSSLTSEDSAVYY	CTIETSQGWFA YW	GQGTLV TVSA
**(8)-10G3**	QVQLQQSGAELVKPGT SVKLSCKAS	GYTLIN YD	INWVRQRPEQGL EWIGW	ILPGDG RT	KYNEKFKGKATLTIDKSSSTAYMQL SRLTSEDSAVYF	CASDSYDNYET YW	GQGTLV TVST
**(8)-8C12**	EVKLEESGGGLVQPGG SMKLSCAAS	GFTFS DAW	MDWVRQSPEKG LEWVAE	IRNKAN NHAT	YYAESVKGRFTISRDDSKISVYLQM NSLRAEDTGIYY	CVVYDYDKEY W	GQGTTL TVSS

Sequence overlap with HY6-F9 (Figure S3). The sequence and structure of this fentanyl-binding antibody was reported by Pancera, *et al.* in 2023.[8] Apart from divergence in the heavy-chain CDR3 between HY6-F9 and all of our mAbs, (**6**)-4C6 differs from HY6-F9 by only one amino acid. Antibodies (**6**)-1C2 and (**8**)-8C12 share chain-dependent CDR sequences with HY6-F9, the former differing by only one residue in the heavy-chain CDR1 and CDR2, and the latter having only three different residues in the light chain CDRs.

Table S3. Closest matches to the CDR sequences of the anti-fentanyl antibody HY6-F9 reported by Pancera and colleagues.[8] Amino acid mismatches are underlined and in red. (*right*) Patterns of fentanyl derivative recognition for the three antibodies discussed here, excerpted from Figure 7.

light chain			
**clone**	**CDR1**	**CDR2**	**CDR3**
HY6-F9	RSSKSLLHSNGITYLY	QMSNLAS	AQNLELPWT
(**6**)-4C6	RSSKSLLHSNGITYLY	QMSNLAS	AQNLELPWT
(**8**)-8C12	RSSKSLLHSDGITYLH	QMSNLAS	VQNLELPWT
(**6**)-1C2 (unrelated)	QNVGTN	KALISAS	QQYNSYPLT
**heavy chain**			
**clone**	**CDR1**	**CDR2**	**CDR3**
HY6-F9	TSGY WN W	YISYSGSTYYNPSLKS	YYGDNYVGAMD Y
(**6**)-4C6	TSGY WN W	YINYSGSTYYNPSLKS	YYCARYPCDGHNCY
(**6**)-1C2	TSGYSWH W	YISYDGSNNYNPSLKG	YYDGATPLTGD Y
(**8**)-8C12 (unrelated)	TFSDAWMDW	VAEIRNKANNHATYYAES	YYCVVYDYDKE Y

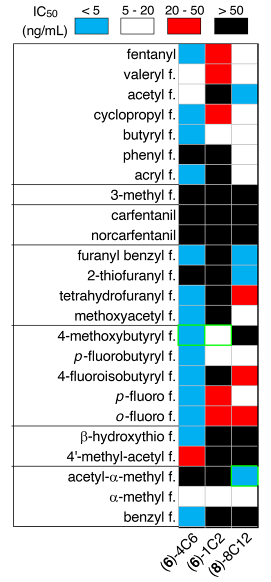

Chemical synthesis and characterization

Synthetic details have been peer-reviewed but are omitted from this document to avoid public dissemination of methods to prepare fentanyl derivatives. This information is available to responsible parties upon request.

References

1. Polonskaya, Z.; Deng, S.; Sarkar, A.; Kain, L.; Comellas-Aragones, M.; McKay, C.; Kaczanowska, K.; Holt, M.; McBride, R.; Palomo, V.; Self, K.; Taylor, S.; Irimia, A.; Mehta, S.R.; Dan, J.M.; Brigger, M.; Crotty, S.; Paulson, J.C.; Wilson, I.A.; Savage, P.B.; Finn, M.G.; Teyton, L., T cells control the generation of nanomolar-affinity anti-glycan antibodies. *J. Clin. Invest.* **2017**, *127*, 1491-1504.
2. Zhao, L.; Kopylov, M.; Potter, C.S.; Carragher, B.; Finn, M.G., Engineering the PP7 Virus Capsid as a Peptide Display Platform. *ACS Nano* **2019**, *13*, 4443-4454.
3. Liu, Z.; Guo, J., NKT-cell glycolipid agonist as adjuvant in synthetic vaccine. *Carbohydr. Res.* **2017**, *452*, 78-90.
4. Chapman, A.P.; Tang, X.; Lee, J.R.; Chida, A.; Mercer, K.; Wharton, R.E.; Kainulainen, M.H.; Harcourt, J.L.; Martines, R.B.; Schroeder, M.; Zhao, L.; Bryksin, A.; Zhou, B.; Bergeron, E.; Bollweg, B.C.; Tamin, A.; Thornburg, N.; Wentworth, D.E.; Petway, D.; Bagarozzi Jr., D.; Finn, M.G.; Goldstein, J.M., Rapid Development of Neutralizing and Diagnostic SARS-COV-2 Mouse Monoclonal Antibodies. *Sci. Rep.* **2021**, *11*, 9682.
5. Bennett, B.; Check, I.J.; Olsen, M.R.; Hunter, R.L., A comparison of commercially available adjuvants for use in research. *J. Immunol. Methods* **1992**, *153*, 31-40.
6. Cribbs, D.H.; Ghochikyan, A.; Vasilevko, V.; Tran, M.; Petrushina, I.; Sadzikava, N.; Babikyan, D.; Kesslak, P.; Kieber-Emmons, T.; Cotman, C.W., Adjuvant-dependent modulation of Th1 and Th2 responses to immunization with β-amyloid. *Int. Immunol.* **2003**, *15*, 505-514.
7. Wharton, R.E.; Casbohm, J.; Hoffmaster, R.; Brewer, B.N.; Finn, M.; Johnson, R.C., Detection of 30 Fentanyl Analogs by Commercial Immunoassay Kits. *J. Anal. Toxicol.* **2021**, *45*, 111-116.
8. Rodarte, J.V.; Baehr, C.; Hicks, D.; Liban, T.L.; Weidle, C.; Rupert, P.B.; Jahan, R.; Wall, A.; McGuire, A.T.; Strong, R.K.; Runyon, S.; Pravetoni, M.; Pancera, M., Structures of drug-specific monoclonal antibodies bound to opioids and nicotine reveal a common mode of binding. *Structure* **2023**, *31*, 20-32. e25.
